# Combined inhibition of class 1-PI3K-alpha and delta isoforms causes senolysis by inducing p21^WAF1/CIP1^ proteasomal degradation in senescent cells

**DOI:** 10.1038/s41419-024-06755-x

**Published:** 2024-05-29

**Authors:** Judith Neuwahl, Chantal A. Neumann, Annika C. Fitz, Anica D. Biermann, Maja Magel, Annabelle Friedrich, Lorenz Sellin, Björn Stork, Roland P. Piekorz, Peter Proksch, Wilfried Budach, Reiner U. Jänicke, Dennis Sohn

**Affiliations:** 1 Laboratory of Molecular Radiooncology, Clinic and Policlinic for Radiation Therapy and Radiooncology, Medical Faculty and University Hospital Düsseldorf, Heinrich-Heine-University Düsseldorf, Düsseldorf, Germany; 2 Experimental Nephrology, Clinic for Nephrology, Medical Faculty and University Hospital Düsseldorf, Heinrich-Heine-University Düsseldorf, Düsseldorf, Germany; 3 Institute of Molecular Medicine I, Medical Faculty and University Hospital Düsseldorf, Heinrich-Heine-University Düsseldorf, Düsseldorf, Germany; 4 Institute of Biochemistry and Molecular Biology II, Medical Faculty and University Hospital Düsseldorf, Heinrich-Heine-University Düsseldorf, Düsseldorf, Germany; 5 Institute of Pharmaceutical Biology and Biotechnology, Heinrich-Heine-University Düsseldorf, Düsseldorf, Germany; 6 Present Address: Functional Microbiome Research Group, Institute of Medical Microbiology, University Hospital of RWTH, Aachen, Germany

**Keywords:** Tumour-suppressor proteins, Senescence, Cell death, DNA damage response

## Abstract

The targeted elimination of radio- or chemotherapy-induced senescent cells by so-called senolytic substances represents a promising approach to reduce tumor relapse as well as therapeutic side effects such as fibrosis. We screened an in-house library of 178 substances derived from marine sponges, endophytic fungi, and higher plants, and determined their senolytic activities towards DNA damage-induced senescent HCT116 colon carcinoma cells. The Pan-PI3K-inhibitor wortmannin and its clinical derivative, PX-866, were identified to act as senolytics. PX-866 potently induced apoptotic cell death in senescent HCT116, MCF-7 mammary carcinoma, and A549 lung carcinoma cells, independently of whether senescence was induced by ionizing radiation or by chemotherapeutics, but not in proliferating cells. Other Pan-PI3K inhibitors, such as the FDA-approved drug BAY80-6946 (Copanlisib, Aliqopa®), also efficiently and specifically eliminated senescent cells. Interestingly, only the simultaneous inhibition of both PI3K class I alpha (with BYL-719 (Alpelisib, Piqray®)) and delta (with CAL-101 (Idelalisib, Zydelig®)) isoforms was sufficient to induce senolysis, whereas single application of these inhibitors had no effect. On the molecular level, inhibition of PI3Ks resulted in an increased proteasomal degradation of the CDK inhibitor p21^WAF1/CIP1^ in all tumor cell lines analyzed. This led to a timely induction of apoptosis in senescent tumor cells. Taken together, the senolytic properties of PI3K-inhibitors reveal a novel dimension of these promising compounds, which holds particular potential when employed alongside DNA damaging agents in combination tumor therapies.

## Introduction

Senescence as a cell fate process is defined as a stable and typically terminal cell cycle arrest that is induced during aging because of telomere shortening or prematurely after oncogene activation or massive DNA damage to prevent mutations and uncontrolled cell growth [1]. On the molecular level, the senescence program is mainly executed by two pathways: activation of the tumor suppressor p53 and its target gene, the cyclin-dependent kinase (CDK)-inhibitor p21^WAF1/CIP1^, or by upregulation of another CDK-inhibitor, p16^INK4A^ [2]. In both cases the inhibition of CDKs and the subsequent absence of phosphorylation of the retinoblastoma protein (Rb) results in senescence induction and the manifestation of its manifold phenotypes, such as massive cell enlargement, increased activity of senescence-associated beta-galactosidase (SABG), and formation of the senescence-associated secretory phenotype (SASP) [3].

The SASP is mainly responsible for the interaction of senescent cells with their surrounding tissue. Its formation results in the secretion of a plethora of cytokines, chemokines, growth factors and extracellular matrix-modifying proteins, ultimately creating an inflammatory microenvironment [4]. Although the essential role of senescence in wound healing processes has been acknowledged [5], its clinical relevance in both tumor as well as non-malignant cells, especially in the context of a radio- or chemotherapy, has been neglected for a long time. However, the chronic accumulation of senescent cells during aging or after a DNA damage-based anti-cancer therapy results in permanently inflamed tissue. Subsequently, the latter becomes non-functional or supports tumor relapse and the generation of therapeutic side effects such as fibrosis (excellently reviewed in [6]).

Recently, groundbreaking research utilizing two different genetically-modified mouse models, the INK-ATTAC [7] and the p16-3MR mouse [5], has demonstrated the impact of cellular senescence on various age-related pathologies, such as tau-dependent neurodegeneration and bone loss, and revealed the clinical benefits of the timely elimination of senescent cells [8]. Unfortunately, they display a very high resistance to apoptosis- or other cell death-inducing reagents [9, 10]. Thereby, the identification of so-called senolytic compounds, which specifically target senescent cells and result in their demise without harming physiologically proliferating cells, is required to successfully translate these discoveries into a treatment regimen usable in patients [11]. First generation senolytics were found by drug repurposing of the naturally-occurring flavonoids fisetin and quercetin, that were either employed alone or in combination with the pan-tyrosine kinase inhibitor dasatanib. However, although this treatment successfully resulted in the elimination of senescent cells under some circumstances [12], the general senolytic properties of these substances could not be confirmed in other reports [13, 14]. Other compounds recently developed, such as the Bcl-2-inhibitor Navitoclax or the FoxO4-DRI-peptide, possess strong senolytic properties and represent promising therapeutic options that are currently tested in clinical trials [11]. Nevertheless, the identification of novel potent senolytics for the future use in cancer treatment, especially in the context of a DNA damage-based and potentially senescence-inducing combinational therapy [15], represents an important endeavor.

We have chosen to analyze an in-house library of natural compounds with regard to their potential senolytic properties towards tumor cells driven into senescence by ionizing radiation (γIR)-induced DNA damage. This library has already been used to identify novel modulators of, among others, autophagy, the DNA damage response or apoptosis [16, 17].

## Results

### Screening of a natural compound library to identify senolytic substances

**Fig. 1:**
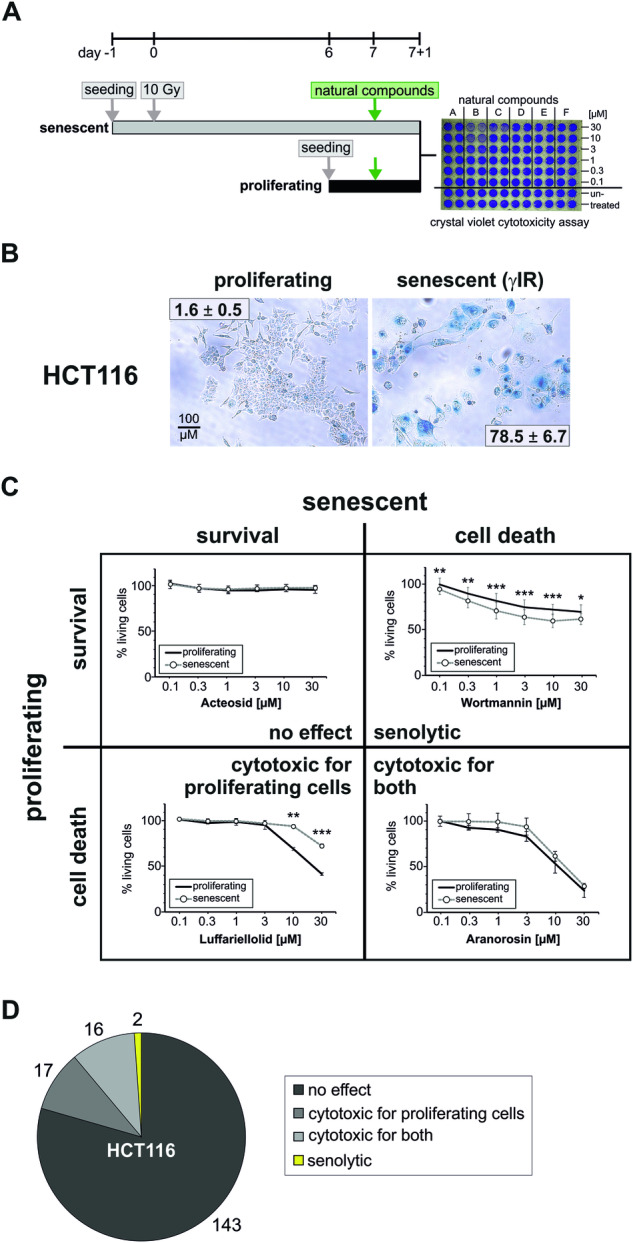
Screening of an in-house library of natural compounds to identify senolytic substances. **A** HCT116 cells were seeded in 96-well plates the day before they were exposed to 10 Gy gamma-irradiation. Seven days later, after the senescent phenotype was fully formed (see **B**), the cells were exposed to the natural compounds in duplicates in concentrations ranging from 30 µM to 100 nM. Proliferating non-irradiated cells were seeded the day before treatment with the natural compounds. 24 h after treatment, cell death was assessed by staining the surviving cells using a crystal violet-based cytotoxicity assay. Each substance was tested in at least three independent experiments. **B** Proliferating or senescent (day 6 after γIR) HCT116 cells treated as described in (**A**), but in a 6-well plate, were stained for senescence-associated beta-galactosidase activity. Positive cells display a strong blue color. All microscopic pictures were taken with the same ×10 magnification (scale bar 100 µM) and are representative for at least three independent experiments. The specified values are the mean percentage of counted blue cells ± SD from these at least three independent experiments (at least 200 to 1000 counted cells per condition and experiment). **C** All compounds were classified based on their cytotoxic effect on proliferating and/or senescent cells. For each class, one exemplary substance is shown. All data shown are the mean ± SD of at least three independent experiments. *p < 0.05, **p < 0.01, ***p < 0.001. **D** Distribution of the 178 tested compounds into four different groups (cytotoxic/non-cytotoxic towards proliferating/senescent cells). Two substances, Kahalalide F and wortmannin, exerted senolytic properties towards HCT116 cells.

Senolytics represent an interesting new approach for both the treatment of aging disorders as well as for the use in anti-cancer-therapy. Therefore, the identification of novel senolytics is an important task, especially because senescent cells often exhibit high resistance towards cell death induction [10]. We employed a crystal violet-based cytotoxicity assay in 96-well microplates to screen an in-house library of natural compounds [16, 17] for its impact on the survival of proliferating and γIR-induced senescent HCT116 colon carcinoma cells (Fig. 1A). Their senescent status six days after γIR was confirmed by staining for senescence-associated beta-galactosidase activity (Fig. 1B). Both proliferating and senescent cells were subsequently treated for 24 h with different doses (from 30 to 0.1 µM) of 178 substances of the library (Fig. 1A). As a negative solvent control, DMSO was used (Supplementary Fig. S1B), whereas we employed the kinase inhibitor staurosporine as a positive control that eliminates both proliferating and senescent cells (Supplementary Fig. S1C).

**Fig. 2:**
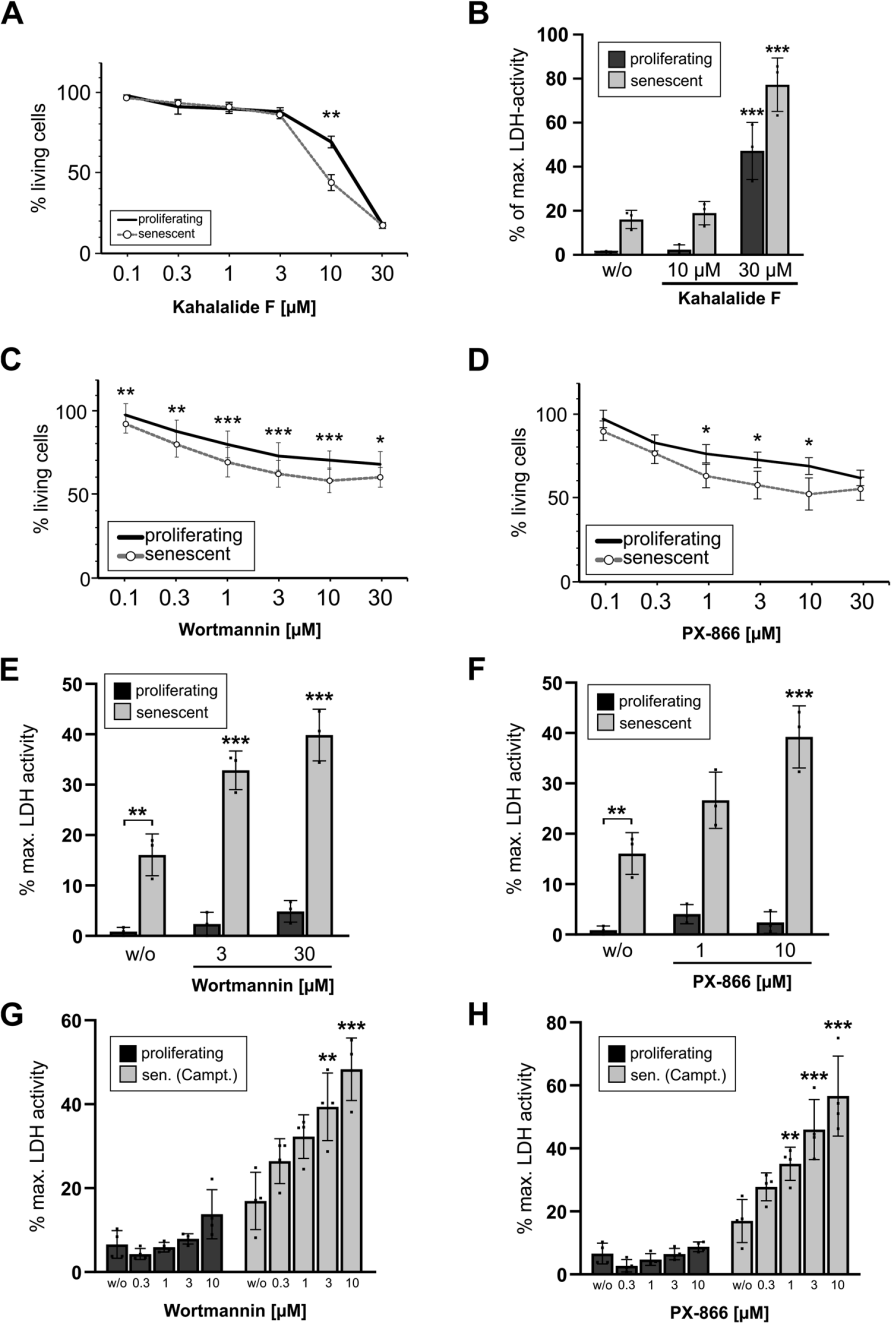
The Pan-class 1-PI3K inhibitor wortmannin and its clinical derivative PX-866 possess senolytic properties towards HCT116 cells. **A** Results for the substance Kahalalide F obtained from the experiments described in Fig. 1. In short, proliferating and senescent (day 7 after γIR) HCT116 cells were treated with different concentrations of Kahalalide F for 24 h before the remaining living cells were quantified by a crystal violet cytotoxicity assay. **B** As an alternative approach to measure cell death, the activity of LDH in the supernatant of cells similarly treated as in (**A**) was quantified. Specific results for the substance wortmannin (**C**) and its clinical derivative PX-866 (**D**) obtained from the experiment described in Fig. 1. Please note that the reduction of % living cells observed after treatment of proliferating HCT116 cells is not because of an increase in cell death, but because of a decrease in proliferation rates. Hence, less cells are stained compared to an untreated control (see also Supplementary Fig. S1D, E and Fig. 2E, F). Asterisks depict the signficance p-value between the amount of living proliferating and senescent cells treated similarly. **E**, **F** In cells treated similar to (**C**, **D**), cell death was alternatively assessed by quantifiying the activity of LDH released into the supernatant. Please note that neither wortmannin (**E**) nor PX-866 (**F**) induce cell death in proliferating cells, in contrast to senescent cells. **G**, **H** Cell death induction of proliferating and senescent (day 7 after camptothecin) HCT116 after treatment for 24 h with the indicated concentrations of wortmannin (**G**) or PX-866 (**H**) by quantifying the activity of released LDH in the cell culture supernatant. All data shown are the mean ± SD of at least three independent experiments. Individual data points are represented in the bar charts by small black squares. If not indicated otherwise, significance asterisks relate to the corresponding control (proliferating/senescent) treated with DMSO (i.e. labeled w/o). *p < 0.05, **p < 0.01, ***p < 0.001.

Most of the substances had no impact on both proliferating or senescent HCT116 cells (‘no effect’, 80.5%). Some substances were cytotoxic solely to proliferating cells (9.5%) or to both proliferating and senescent cells (9%) (Fig. 1C, D, Supplementary Table 1). Only two compounds, Kahalalide F and wortmannin, exhibited a senolytic potential (1%), *i.e*. they were significantly more cytotoxic towards senescent cells (Figs. 1C, D and 2A, C). The cytoxicity assay demonstrated that Kahalalide F posesses a very small effective concentration range around 10 µM in which it selectively killed senescent cells (Fig. 2A). This was confirmed when measuring the activity of released lactate dehydrogenase (LDH) in the cell culture supernatant after treatment as a marker for universal cell death. Although a huge proportion of the senscent cells were killed by Kahalalide F, a substantial amount of proliferating cells was also eliminated (Fig. 2B). Thereby, because of this too small therapeutic dose window, Kahalalide F cannot be categorized to be a purely senolytic compound and it would be difficult to establish its use in a clinical setting.

### The Pan-class 1-PI3K inhibitor wortmannin and its clinical derivative PX-866 exhibit senolytic properties

**Fig. 3:**
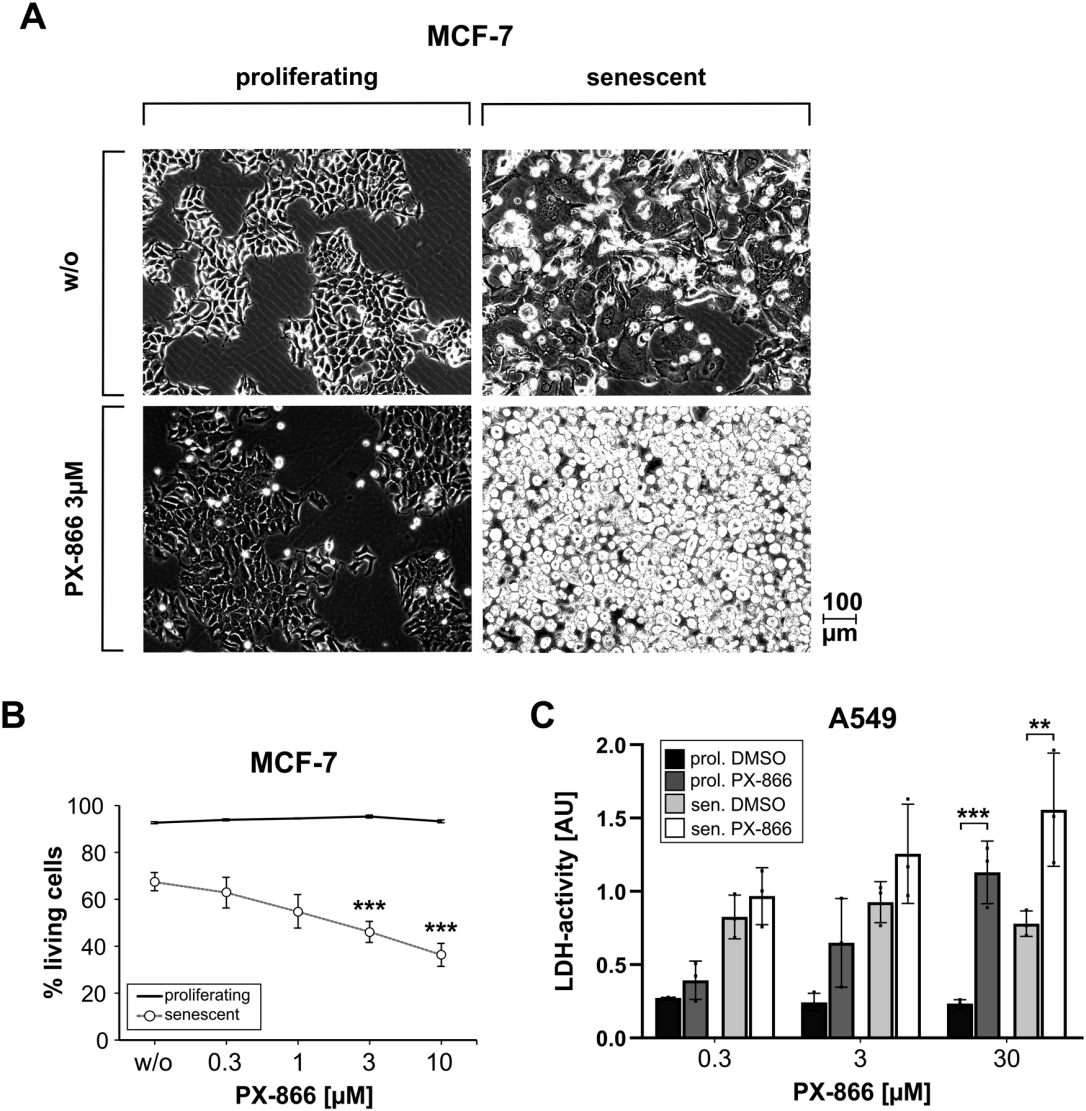
The PI3K inhibitor PX-866 constitutes a senolytic compound for MCF-7 and A549 tumor cells. **A** Proliferating and senescent (day 7 after γIR) MCF-7 cells were treated with PX-866 for 24 h. Subsequently, micrographs of the samples without prior aspiration were taken with 10x magnification. In the lower right picture (PX-866 treated senescent cells) the strong accumulation of dead detached cells can be observed. The shown micrographs are representative for at least three independently performed experiments. **B** Proliferating and senescent (day 7 after γIR) MCF-7 cells were treated for 24 h with the specified concentrations of PX-866. Subsequently, cell death induction was quantified flow cytometrically by the determination of PI uptake-negative cells. **C** Proliferating and senescent (day 7 after γIR) A549 cells were treated with different concentrations of PX-866 for 24 h. Subsequently, cell death induction was quantified by measuring released LDH activity in the cell culture supernatant. Data shown in (**B**, **C**) are the mean ± SD of at least three independent experiments. Individual data points are represented in the bar charts by small black squares. If not indicated otherwise, significance asterisks relate to the corresponding control (proliferating/senescent) treated with DMSO (i.e. labeled w/o). *p < 0.05, **p < 0.01, ***p < 0.001.

Wortmannin possesses a very short half life in cell culture media. Therefore, we additionally included its more stable, already clinically-tested derivative PX-866 (also called Sonolisib) [18] in all verification experiments. Both compounds resulted in less survival of senescent HCT116 cells over a wide dose range. However, proliferating cells were also detected at reduced levels after such a treatment, apparantly indicating cell death, based on the results from the crystal violet cytotoxicity assay (Fig. 2C, D). However, when trying to verify this observation by measuring extracellular LDH activity as a different cell death marker, only the senescent cells demonstrated a dose-dependent release of LDH into the supernatant that was not detected in proliferating cells (Fig. 2E, F). Upon visual inspection we concluded that a treatment of proliferating cells with wortmannin or PX-866 did not induce more cell death (no detachment of dead cells) but led to smaller overall cell numbers, most probably because of reduced proliferation rates (data not shown, see also Fig. 3A for a similar observation in MCF-7 breast cancer cells). This resulted in a decreased staining of proliferating cells in the crystal violet cytotoxicity assay (Fig. 2C, D), which is based on the detection of living, still adherent cells. Thus, in such an assay, a sample consisting of proliferating cells treated with PX-866 mistakingly appears to be partially dying. In concordance with the observed reduced cell numbers, PX-866 treatment of proliferating cells resulted in a partial arrest of the cells in the G_1_-phase and a strong decrease of S-phase cells, both in HCT116 and MCF-7 cells (Supplementary Fig. S1D, E). It is important to note, that because senescent cells are not growing (respectively cycling) anymore, this effect does not impact the measurement of PX-866 cytotoxicity towards senescent cells. The PI3K-inhibitors wortmannin and PX-866 decrease the division rate of proliferating cells, but they induce cell death in senescent cells, thereby making them bona fide senolytic compounds.

HCT116 cells driven into senescence by using camptothecin [15] were also efficiently eliminated in a dose-dependent manner with wortmannin and PX-866 (Fig. 2G, H). Similarly, γIR-induced senescent MCF-7 cells (Supplementary Fig. S1A) were specifically killed by PX-866 as demonstrated by microscopic imaging (Fig. 3A, detached dying cells in lower right panel) and flow-cytometric analysis of propidium iodide uptake (Fig. 3B). We expanded our analyses to an additional tumor entity and investigated the lung carcinoma cell line A549 (Supplementary Fig. S1A). Again, PX-866 was able to induce cell death in senescent cells, however, it also eliminated proliferating cells (Fig. 3C). In this case, PX-866 can be considered to be mainly senolytic, but is also cytotoxic towards proliferating A549 cells.

Taken together, the Pan-PI3K-inhibitor PX-866 can be classified as a novel senolytic compound in cell lines of different tumor entities that were driven into senescence by both radio- or chemotherapy.

### PX-866 induces apoptosis specifically in senescent cells

**Fig. 4:**
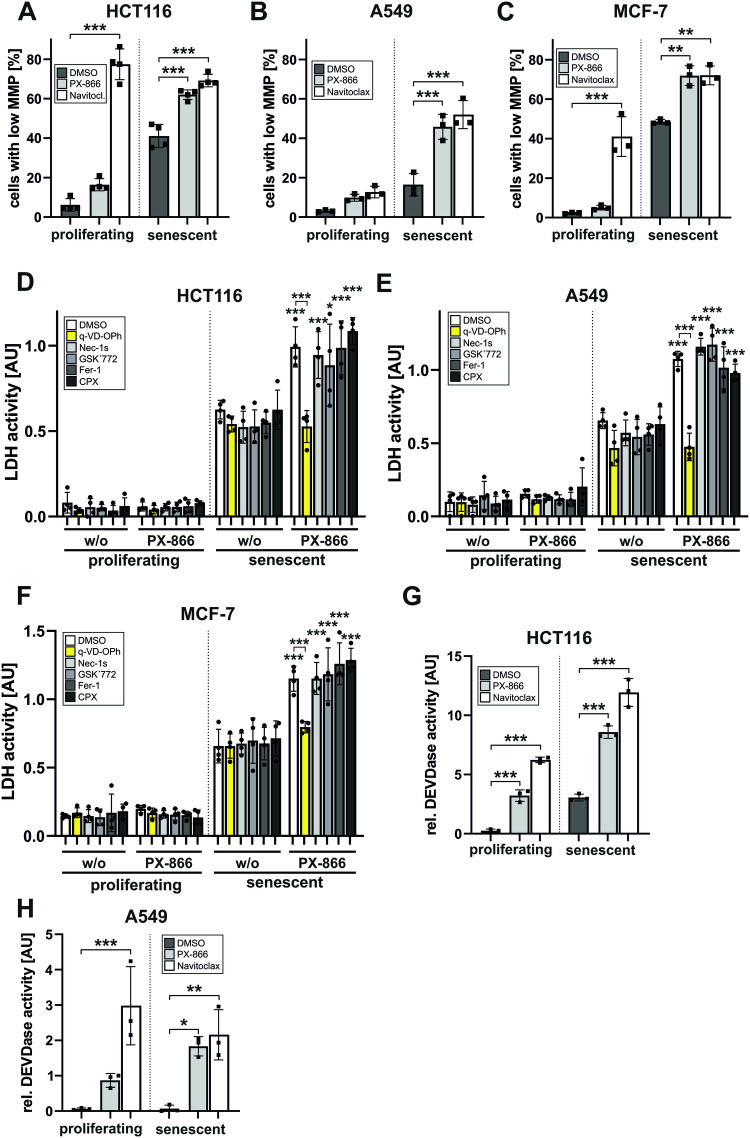
PX-866 induces apoptosis specifically in senescent cells. **A**–**C** Flow cytometrical determination of the mitochondrial membrane potential (MMP) of proliferating and senescent (day 7 after γIR) HCT116 (**A**), A549 (**B**) and MCF-7 (**C**) cells after 24 h treatment with PX-866 (**A**: 10 µM; **B**, **C**: 30 µM) or Navitoclax (10 µM). **D**–**F** Proliferating and senescent (day 7 after γIR) HCT116 (**D**), A549 (**E**) and MCF-7 (**F**) cells were exposed to PX-866 (**D**, **E**: 10 µM; **F:** 30 µM) together with the apoptosis inhibitor q-VD-OPh (in yellow), the necroptosis inhibitors Nec-1s and GSK’872, or the ferroptosis inhibitors Fer-1 and CPX (all 10 µM). After 24 h, LDH activity in the cell culture supernatant was determined as a marker for cell death. Only a co-treatment with q-VD-Ph inhibited the PX-866-mediated increased death of senescent cells. Significance indicators above the bars are comparing the sample of senescent cells of the corresponding inhibitor with or without PX-866 treatment. The exception is the sample of PX-866 and q-VD-OPh treated senescent cells which is not significantly altered by PX-866 treatment. This sample is significantly different to a treatment with PX-866 without q-VD-OPh (indicated by brackets). **G**, **H** Measurement of caspase-3-like (DEVDase) activity in cellular extracts of proliferating and senescent (day 7 after γIR) HCT116 (**G**) and A549 (**H**) cells after an additional treatment with PX-866 (**G**: 10 µM; **H**: 30 µM) or Navitoclax (10 µM). Data shown are the mean ± SD of at least three independent experiments. Individual data points are represented in the bar charts by small black squares. *p < 0.05, **p < 0.01, ***p < 0.001.

To identify the senolytic mechanism induced by PI3K-inhibition, we determined the amount of cells characterized by a loss of the mitochondrial membrane potential (MMP) after treatment with PX-866 using flow cytometry. Induction of senescence upon γIR exposure resulted in a certain ‘background’ level of HCT116 and MCF-7 cells with a decreased MMP (Fig. 4A, C). However, this was not detectable in γIR-treated A549 cells (Fig. 4B). Such a strong reduction of the MMP was previously observed [19, 20] and is described as one characteristic of senescent cells [6] that alone does not serve as a reliable indicator for apoptotic cell death. As a positive control, we employed a potent inhibitor of anti-apoptotic Bcl-2 family members, Navitoclax, which beside its original function to target proliferating tumor cells has also been described to act as a powerful senolytic and apoptosis-inducing compound [11, 21]. Concordantly, in all three tested cell lines, Navitoclax induced loss of the MMP in an increased number of both proliferating and senescent cells (Fig. 4A–C), except for proliferating A549 cells, which seem to be resistant to such a treatment (Fig. 4B). PX-866, however, only affected senescent cells (Fig. 4A-C), but similar to a treatment with Navitoclax.

In a next step, we employed several inhibitors targeting apoptosis (q-VD-OPh), necroptosis (Nec-1s and GSK’872) or ferroptosis (Fer-1 and CPX) together with PX-866. All these cell death pathways have been described to be potentially induced after γIR [22, 23]. However, only the caspase-inhibitor q-VD-OPh (bars marked in yellow) clearly and significantly inhibited the PX-mediated death of senescent cells, as shown by a reduced activity of LDH released into the supernatant (Fig. 4D–F). This was demonstrated not only in HCT116 and A549 (Fig. 4D, E) but also in MCF-7 cells (Fig. 4F). On the first look this seems surprising because MCF-7 cells lack expression of the main executioner caspase-3 and therefore do not display typical apoptotic features [24]. However, MCF-7 cells are still susceptible to diverse apoptotic stimuli as they still express the executioner caspase-6 and -7 and undergo cell death without displaying apoptotic markers such as membrane blebbing or DNA fragmentation [25].

As a final evidence for apoptosis induction caspase-3-like activities were determined in the cellular extracts of proliferating and senescent cells treated with PX-866 or Navitoclax. Whereas Navitoclax induced such activites in both proliferating and senescent HCT116 and A549 cells, PX-866 induced caspase-3 activation mainly in senescent cells (Fig. 4G, H). However, for both Navitoclax and PX-866 and in concordance with the observed loss of the MMP (Fig. 4A, B), caspase activity in senescent A549 cells was much lower than in senescent HCT116 cells (Fig. 4G, H).

In summary, these data demonstrate that PX-866 induces apoptosis specifically in senescent HCT116, A549 and MCF-7 cells to a similar extent as Navitoclax.

### Treatment of senescent cells with PX-866 results in reduced cyclin D1 levels and proteasomal degradation of p21^WAF1/CIP^

**Fig. 5:**
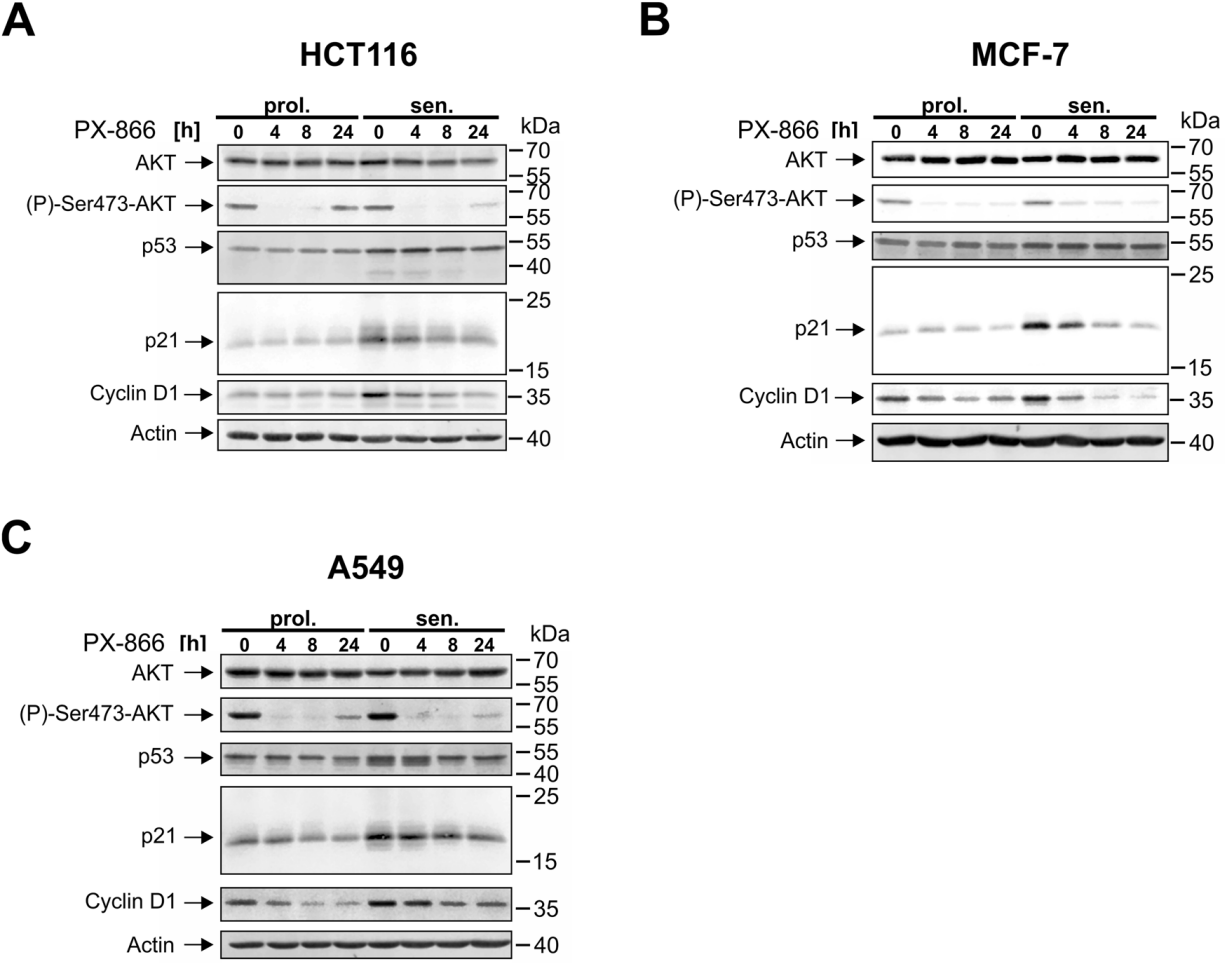
Treatment with PX-866 results in a decrease of cyclin D1 and p21 protein levels in senescent cells. Proliferating and senescent (day 7 after γIR) HCT116 (**A**), MCF-7 (**B**) and A549 (**C**) cells were treated with PX-866 (10 µM) for the indicated times before they were harvested and subjected to western blot analysis. Please see also Fig. 6D (HCT116) and 6F (MCF-7) for the densitometric analysis of p21 protein levels in similarly treated senescent cells with PX-866. The blots shown are representative for at least three independent experiments.

On the molecular level, DNA damage-induced senescence is characterized by the accumulation of the tumor suppressor p53 and its target gene, the CDK inhibitor p21^WAF1/CIP1^. Both proteins are essential for the initiation and maintenance of cellular senescence after DNA damage [26]. In addition, an increase in cyclin D1 expression is also often observed [27]. This molecular senescent phenotype can be demonstrated after treatment with γIR (Fig. 5A–C) or camptothecin (Supplementary Fig. S2A–C) in all three cell lines investigated (HCT116, MCF-7, and A549). Treatment of both proliferating and senescent cells with PX-866 for up to 24 h resulted in the expected loss of AKT phosphorylation at serine 473, which is required for full activation of AKT by PI3Ks [28]. Interestingly, PX-866 also strongly reduces the expression of cyclin D1 and p21 in a time-dependent manner, correlating with the induction of cell death in senescent cells. In HCT116 and A549 (Fig. 5A, C, Supplementary Fig. S2A, C) an additional decrease in p53 levels can be observed, but not in MCF-7 cells (Fig. 5B and Supplementary Fig. S2B).

**Fig. 6:**
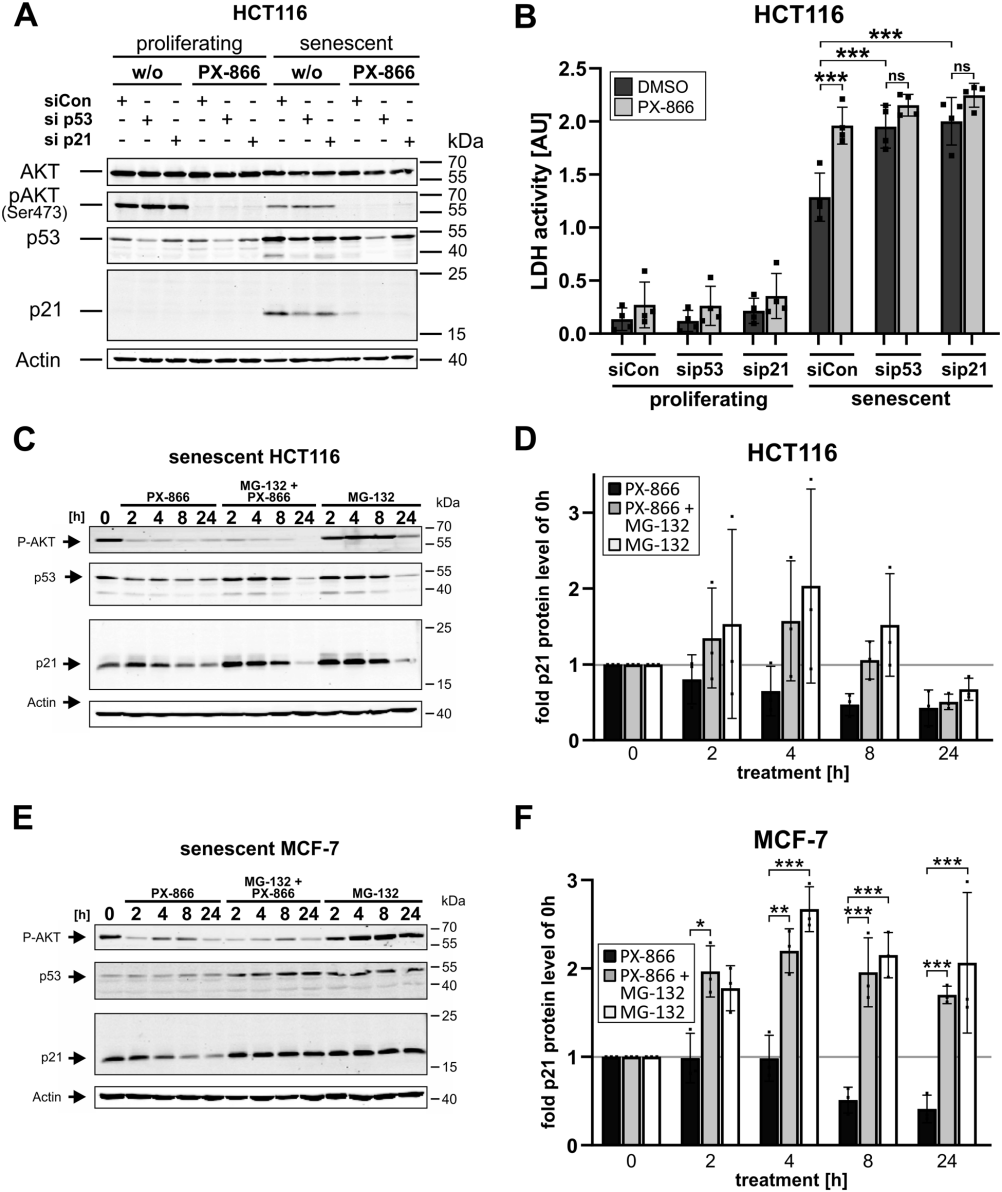
PX-866 induces proteasomal degradation of p21 in senescent cells. **A**, **B** Proliferating and senescent (day 7 after γIR) HCT116 cells were transfected with a non-targeting control siRNA or siRNAs directed against p53 or p21. 48 h after transfection, the cells were additionally treated with PX-866 (3 µM) for another 24 h. Cell death was assessed by quantifying LDH activity released into the cell culture supernatant (**B**), whereas inhibition of AKT phosphorylation and the knockdown of p53 or p21 protein expression was verified by western blotting (**A**). **C**–**F** Senescent (day 7 after γIR) HCT116 (**C**, **D**) or MCF-7 (**E**, **F**) cells were treated with either 3 µM PX-866, 10 µM MG-132 or a combination of both. At the indicated times, the cells were harvested and the cell extracts subjected to western blot analysis (**C**, **E**). The blots shown are representative of at least three independent experiments. The expression of p21 was quantified densitometrically using the Image Studio (Ver. 5.2) software (LI-COR). Relative p21 levels normalized to the no treatment sample (also indicated by a gray line) were plotted (**D**, **F**). Data shown are the mean ± SD of at least three independent experiments. Individual data points are represented in the bar charts by small black squares. ns not significant *p < 0.05, **p < 0.01, ***p < 0.001.

To clarify if the observed loss of p21 expression is the reason why PX-866 induces death of senescent cells, we performed siRNA-transfection experiments specifically targeting p53 or p21 expression in HCT116 cells. Both siRNAs efficiently reduced the expression levels of the respective target proteins, and the siRNA directed against p53 expectedly also resulted in reduced amounts of the p53 target gene p21 (Fig. 6A). Unfortunately, the siRNA transfection procedure with a non-targeting control siRNA resulted in increased background cell death in the senescent cells on its own. Nevertheless, both p53 and p21 siRNAs still induced an additional significant increase in death of senescent cells, as demonstrated by the release of LDH into the cell culture supernatant, whereas the proliferating cells were not affected (Fig. 6B). This indicates that p21 expression is required for maintaining the senescent state, and that in the absence of p21 protein expression, senescent cells undergo cell death. In addition, whereas cell death of control siRNA transfected cells could be significantly amplified by the use of PX-866, this treatment had no synergistic impact on p53- or p21-siRNA transfected cells (Fig. 6B). In conclusion, loss of p21 expression after PX-866 treatment is responsible for the observed death of senescent cells.

This conclusion is in agreement with other reports demonstrating that the persistent expression of p21 is required for the survival of senescent cells [19, 29, 30]. Therefore it is highly likely that the observed decrease of p21 expression in senescent cells is responsible for the PX-866-induced cell death. PI3K and their downstream kinases (e.g. AKT) are known to phosphorylate p21 at several residues, thereby regulating its cellular location and protein stability [29, 31, 32]. In addition, p21 is also a substrate for other PI3K downstream kinases such as PKCzeta [29], which is activated by PDK1 and GSK3-beta [33], which is phosphorylated and inhibited by AKT [34]. Thus, several kinases participitating in the PI3K signaling pathway regulate p21 protein stability by phosphorylation in both a positive and a negative manner. We therefore speculated that the PI3K-inhibitor PX-866 induces an increase in the proteasomal degradation of p21. Consistent with this, the proteasomal inhibitor MG-132 rescued, at least for the first 8 h of treatment at minimum, the PX-866-mediated loss of p21 protein levels in senescent HCT116 (Fig. 6C, D) and MCF-7 cells (Fig. 6E, F).

In summary, inhibition of PI3Ks by PX-866 results in an increased proteasomal degradation of p21 that leads to apoptosis induction.

### Inhibition of Pan-class 1-PI3Ks is sufficient to eliminate DNA damage-induced senescence cells

We wanted to verify that the observed senolytic potential of PX-866 depends on its desired molecular function, *i.e*. the inhibition of PI3Ks, and not because of unknown unspecific off-target effects. We therefore employed several other inhibitors targeting PI3K class I and III, single class I isoforms or downstream kinases. Our selection was focused on compounds that have already been approved for treatment in patients. Their successful application at the used concentrations was verified by detecting the inhibition of AKT Ser473 phosphorylation (Supplementary Fig. S3). We subsequently analyzed cell death induction by measuring the release of LDH into the supernatant.

**Fig. 7:**
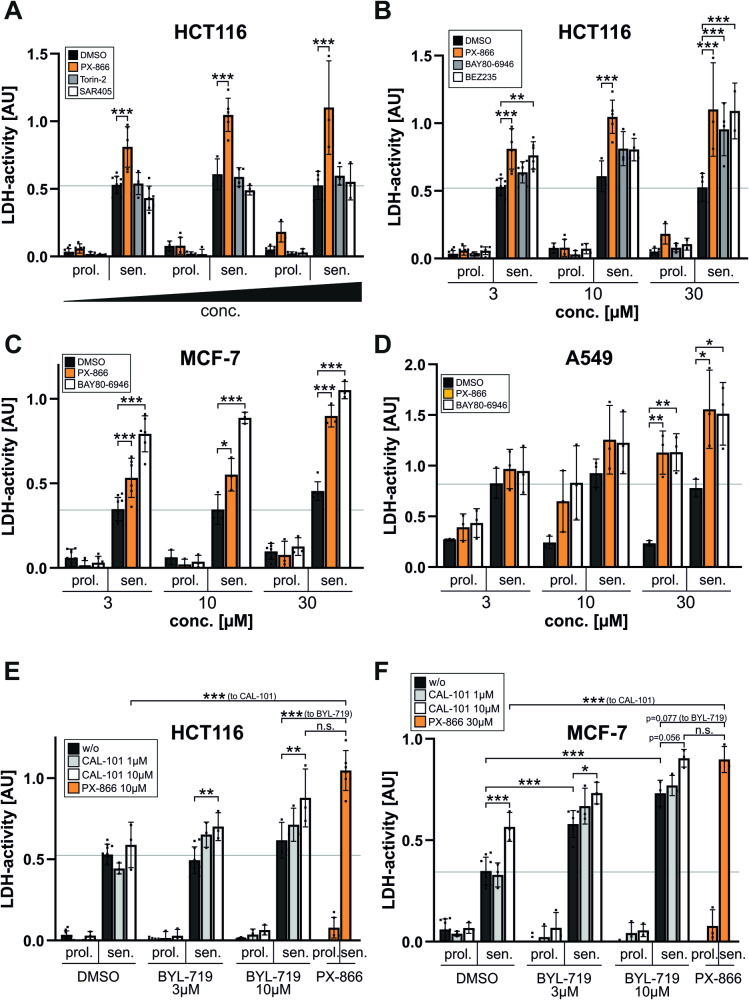
Inhibition of the alpha/delta isoforms of PI3K is required to induce cell death of senescent tumor cells. Proliferating and senescent (day 7 after γIR) HCT116 (**A**, **B**, **E**), MCF-7 (**C**, **F**) and A549 (**D**) cells were treated with PX-866 (3, 10 and 30 µM, orange bars in all panels) or other inhibitors for 24 h before cell death induction was quantified by determining released LDH activity in the cell culture supernatant. In (**A**), autophagy-modulating inhibitors such as Torin-2 (0.1, 0.5 and 1 µM, targets mTOR) and SAR405 (1, 5 and 10 µM, targets Vps34, the only class III PI3K) were tested in addition to PX-866. In (**B**), the dual Pan-PI3K/mTOR inhibitor BEZ235 was analyzed. In **B**–**D**, the pan-PI3K inhibitor BAY80-6946 was employed. In (**E**, **F**), the PI3K-alpha-inhibitor BYL-719 and the PI3K-delta-inhibitor CAL-101 were used either alone or together in two different concentrations (3 and 10 µM for BYL-719, 1 and 10 µM for CAL-101). Please note, that for a better comparison the data bars for the solvent control (DMSO) as well as for PX-866 (in orange) are included as a reference in all graphs. In a similar manner, the results for A549 treated with DMSO and PX-866 (**D**) is also shown in Fig. 3C. A gray line was used in all panels to demonstrate the level of released LDH activity in senescent cells without any other treatment. The data shown in all panels are the mean ± SD of at least three independent experiments. Individual data points are represented in the bar charts by small black squares. n.s. not significant, *p < 0.05, **p < 0.01, ***p < 0.001.

We started to investigate if inhibition of the class III PI3K Vps34 or the mTOR signaling pathway results in a similar senolytic response as a treatment with PX-866. However, neither the Vps34 inhibitor SAR405 nor the mTOR inhibitor Torin-2 induced cell death in senescent HCT116 cells (Fig. 7A, PX-866 shown as orange bar for comparison). This indicates that PI3K class I signaling is required for the survival of the senescent cells. Concordingly, when the Pan-PI3K inhibitors BEZ235 (Dactolisib; targets also mTOR and not yet approved for treatment) and BAY80-6946 (Copanlisib, Aliqopa®; approved for treatment of follicular lymphoma) were applied, similar death rates of senescent HCT166 cells compared to the treatment with PX-866 (Fig. 7, orange bar in all panels) were detected (Fig. 7B). Proliferating cells were not affected by either treatment. BAY80-6946 also displayed similar or even better efficacies towards senescent MCF-7 or A549 cells (Fig. 7C, D). As observed after a treatment with PX-866 (Figs. 3C/7D), A549 cells were already sensitive towards BAY80-6946 (Fig. 7D) in their proliferative state, demonstrating that they are generally susceptible to PI3K inhibitor-mediated cell death. However, senescent A549 cells are killed even more efficiently than the proliferating cells. In conclusion, Pan-PI3K inhibition efficiently induces the death of senescent cells of several different tumor entities.

BAY80-6946, which is more selective towards the alpha and delta isoforms [35], had the biggest impact of all tested Pan-PI3K inhibitors. Because the use of isoform specific-inhibitors is more desired to reduce the occurrence of therapeutic side effects [36], we wanted to investigate if inactivation of defined PI3K class I isoforms is sufficient for the observed senolytic effect. Therefore, we employed BYL-719 (Alpelisib, Piqray®; approved for breast cancer treatment) and CAL-101 (Idelalisib, Zydelig®; approved for treatment of CLL and follicular lymphoma), that specifically target the class 1 PI3K alpha or delta isoforms, respectively. Interestingly, neither of these two inhibitors alone was able to induce cell death of senescent HCT116 (Fig. 7E) and MCF-7 (Fig. 7F) cells as efficiently as PX-866. BYL-719, which is approved for the treatment of breast cancer, had a partial effect on MCF-7 cells when used solely (Fig. 7F). However, when they were applied together, they achieved similar death rates of senescent cells as seen for a treatment with PX-866. This demonstrates that neither the inhibition of the alpha nor of the delta isoform alone is sufficient for senolysis. Both isoforms have to be inhibited to efficiently kill senescent cells, either by using a pan-inhibitor such as PX-866 or by employing a combination of isoform-specific inhibitors.

### Senescent primary fibroblast and epithelial cells are resistant to PX-866 or BAY80-6946 induced senolysis

**Fig. 8:**
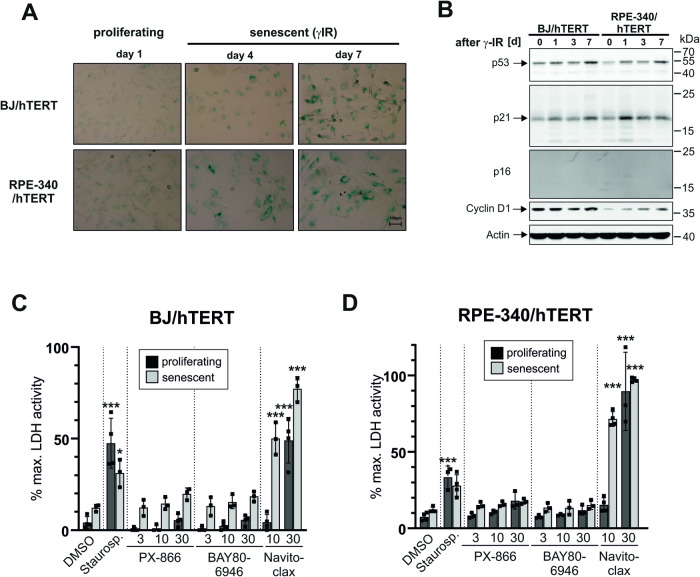
Primary senescent fibroblast and epithelial cells are resistant to PI3K-inhibitor-induced senolysis. **A** The immortalized primary fibroblast BJ/hTERT and epithelial RPE-340/hTERT cell lines were exposed to 20 Gy γIR and senescence induction was determined by staining for senescence-associated beta-galactosidase activity at day 4 and day 7 following irradiation. Senescent cells display a strong blue color. Microscopic pictures were taken with the same 10x magnification (scale bar 100 µM in the lower right panel) and are representative of at least three independent experiments. **B** Western blot analyses of the expression of the indicated proteins in BJ/hTERT and RPE-340/hTERT cells at one, three and seven days after 20 Gy γIR. The blots shown are representative of at least three independent experiments. **C**, **D** Proliferating and senescent (day 7 after γIR) BJ/hTERT (**C**) and RPE-340/hTERT (**D**) cells were exposed to the indicated concentrations of the PI3K inhibitors PX-866 and BAY80-6946, the senolytic compound Navitoclax, and the pan-kinase inhibitor staurosporine (3 µM) as a positive control. After 24 h, LDH activity in the cell culture supernatant as a marker for cell death was determined. The data shown are the mean ± SD of at least three independent experiments. Individual data points are represented in the bar charts by small black squares. Significance indicators relate to the corresponding control (proliferating/senescent) treated with DMSO. *p < 0.05, **p < 0.01, ***p < 0.001.

Our data demonstrate that PI3K-inhibitors efficiently eliminate therapy-induced senescent tumor cells. To evaluate the specificity of this senolytic effect towards transformed cells as compared to benign cells, we employed the primary immortalized BJ/hTERT fibroblast and RPE-340/hTERT epithelial cell lines [37] that do not become replicatively senescent because of constitutive hTERT expression. However, hTERT does not protect cells from premature DNA damage-induced senescence [38, 39]. In concordance with this literature, exposure to 20 Gy γIR resulted in strong appearance of cells positive for the activity of senescence-associated beta-galactosidase over the course of 7 days (Fig. 8A). On the molecular level, and similar to the response of the senescent tumor cell lines (Fig. 5), the protein levels of the tumor suppressor p53 and the senescence markers p21 and cyclin D1 were induced (Fig. 8B). Another CDK-inhibitor, p16, which is primarly responsible for replicative senescence, was not detected. These results demonstrate that the primary cell lines become senescent at day 7 after γIR exposure.

In both proliferating and senescent BJ/hTERT (Fig. 8C) and RPE-340/hTERT (Fig. 8D) cells, staurosporine and Navitoclax efficiently induced cell death. At lower concentrations (3 µM), Navitoclax only mediated senolysis, at higher concentrations (10 µM) it killed both proliferating and senescent cells. In contrast, neither PX-866 nor BAY80-6946 had any impact on both cell lines in all tested concentrations (Fig. 8C, D). In contrast to the tumor cell lines analyzed (Fig. 7), both proliferating and senescent primary cells were fully resistant towards treatment with PI3K inhibitors.

## Discussion

Unlocking the potential of senolytics for a clinical application could be a game changer for the treatment of a multitude of age-associated pathologies as well as for anti-cancer therapy and its potential side effects. However, the identification of specific and potent senolytic compounds is one big obstacle for this approach. Therefore, we tested an in-house library of natural compounds for its senolytic property and identified the PI3K class I inhibitor wortmannin and its clinical derivative PX-866 as substances specifically killing several senescent cancer cell lines by apoptosis. Subsequent experiments demonstrated that single isoform-specific inhibitors were not sufficient to eliminate senescent cells. Instead, simultaneous inhibition of the alpha and delta PI3K isoforms is minimally required for death induction of senescent cells. In addition, senescent primary fibroblast and epithelial cells were resistant towards PI3K-inhibition mediated senolysis. This represents an important property of PI3K inhibitors for a potential in vivo application, since the aim is to selectively eliminate senescent tumor cells.

The functional PI3K signaling heterodimeric complex is formed by association of a catalytic subunit (alpha-delta) with a receptor subunit (p85 & receptor tyrosine kinases (RTKs) or p87/101 & G-protein-coupled receptors (GPCRs)). Both the alpha and the delta isoform of PI3K solely associate with the p85 subunit and reside downstream of RTK activation [40]. In our hands, simultaneous inhibition of both the PI3K alpha and delta isoform leads to death of senescent cells. It is tempting to speculate that factors contained in the SASP induce RTK activation that could be required for the maintenance of the senescent status of the cells. Concordantly, the SASP was already reported to induce or enhance senescence in a paracrine and autocrine manner [6]. In this regard, the unspecific first generation senolytics Dasatanib, Quercetin and Fisetin inhibit, among many other targets, also RTK/PI3K signaling [11]. Pan-PI3K-class I-inhibitors or the combination of PI3K alpha/delta inhibitors could represent a more direct and effective approach with less side effects. Furthermore, all herein tested PI3K inhibitors possess a similar senolytic impact although they are structurally different (albeit all are ATP-analogs) [41], demonstrating the specificity of this approach. Potential off-target effects on other kinases can therefore be excluded.

Together with p53, PI3K is one of the most often mutated signaling pathway in cancer [42]. The senolytic effect of PI3K class I inhibition is in so far unexpected as such compounds are proposed or are already in use for anti-cancer therapies against non-senescent cancer cells [36]. Indeed, we observed an induction of cell death in non-irradiated proliferating A549 cells and both HCT116 and MCF-7 cells displayed a slightly decreased proliferation rate in the presence of pan-PI3K inhibition. However, the observed killing impact on senescent cells was much stronger. Of the herein used PI3K-inhibitors, BAY80-6946, BYL-719, and CAL-101 are already approved from the FDA for cancer treatment and are also advanced in clinical trials for other tumor entities. One could speculate that the observed clinical efficacy of BAY80-6946 and potentially BYL-719, which had a partial senolytic impact on MCF-7 breast cancer cells, is not solely based on their general cell death-inducing and proliferation-decreasing capabilities, but that their so far unknown senolytic function further supports their anti-cancer properties. Thus, it might make sense to use these substances in combination therapies subsequent to a DNA damage-based radio- or chemotherapy in order to eliminate the resulting portion of senescent cells that have escaped primary therapy. In this regard, PI3K inhibitors have already been classified as so-called radiosensitizers that increase the impact of a radiation therapy on cancer cells when given beforehand or concomitantly [43–45]. Inhibition of the DNA damage sensor proteins ATM and DNA-PKcs was proposed to be the underlying molecular mechanism [44, 46]. In our case, however, the PI3K-inhibitor application is performed seven days after radiation treatment. This time period is likely too long for the observed effects to be attributed to radiosensitizing and a decrease in DNA damage recognition and repair. In addition, PX-866-mediated cell death also occurs in cells driven into senescence by camptothecin. Interestingly, whereas inhibition of PI3K alpha isoform was reported to exhibit a radiosensitizing impact, inhibition of the beta isoform did not [47]. For future potential clinical applications the chronological order of combination therapies with pan- or a combination of isoform specific PI3K inhibitors needs to be carefully evaluated, but could be crucial for unlocking their senolytic potential [48].

On the molecular level, pan-PI3K-class I-inhibition resulted in an increased proteasomal degradation of p21 that lead to apoptosis induction of senescent cells. One very prominent PI3K substrate, AKT (protein kinase B), phosphorylates and thereby enhances the p53 inhibiting potential of MDM2 [49]. Furthermore, AKT also directly phosphorylates p21 at several residues (e.g. Thr145, Ser146) [29, 50] and thus regulates its protein stability and localization [32, 50]. Although this would provide a convincing explanation for our observation that p21 degradation is increased in the presence of PI3K inhibitors, there were conflicting reports on the exact consequences of these p21 modifications [29, 31]. However, in our cellular system AKT seems to have no or only a partial impact on the senolytic effect of PI3K class I inhibition. Treatment with the PI3K-alpha inhibitor BYL-719, similar to PX-866 and BAY80-6946, resulted in full inactivation of AKT, but it only efficiently induced death of senescent cells in concert with the PI3K-delta inhibitor CAL-101. Thereby, inhibition of AKT phosphorylation alone is not the crucial step for the induction of senolysis. Most likely the activity of one of the many other direct or indirect PI3K downstream targets such as the PKC/RAS- or Rac/Rho/ROCK signaling pathways, PDK1/SGK3 [51], BTK and others [52] is required to explain the full senolytic impact of pan-PI3K inhibition. Some of these targets have been reported to associate with p53 signaling pathways and/or p21 expression directly [29, 53, 54] and thereby resemble potential candidates how PI3K-inhibition leads to less p21 protein.

Reduced p21 levels in senescent cells, either by PX-866 treatment or by siRNA transfection, resulted in the induction of apoptosis, as demonstrated by inhibition of PX-866 induced senolysis by the caspase-inhibitor q-VD-OPh. It was already reported that p21 actively inhibits caspase activation [29–31], which is one reason why senescent cells survive in the presence of an unresolved DNA damage response [6, 30]. This would also explain the fast kinetic of a strong apoptosis induction after a 24 h treatment period with PI3K-inhibitors. In addition, reduced p21 levels are not sufficient to maintain the cellular senescence status. This could potentially result in a cell cycle re-entry of the highly-damaged cells and, consequently, the induction of mitotic catastrophe [23].

Although the PI3K signaling pathway has already been proposed to be important for senescence maintenance, this is the first report demonstrating that direct inhibition of two PI3K class I isoforms, alpha and delta, is sufficient to induce apoptosis-mediated senolysis. Isoform-specific inhibitors of PI3Ks are heavily investigated for clinical use and represent a major breakthrough for anti-cancer therapy [36]. Our data demonstrate that the combination of a senescence-inducing therapy together with such PI3K inhibitors in form of a ‘one-two-punch’ constitutes a promising new approach to improve cancer treatment [11, 55].

## Materials and methods

### Cell lines, treatments and reagents

HCT116 cells [56] were cultured in McCoy’s 5A GlutaMAX-Medium (Gibco, Thermo Fisher Scientific, Waltham, MA, USA), whereas the breast cancer cell line MCF-7 [24] was propagated in RPMI-1640 GlutaMAX (Gibco). The lung carcinoma cell line A549 was cultivated in F-12K-Medium (Gibco). All three tumor cell lines are expressing p53 wild-type protein and are deficient for p16 expression. The immortalized primary fibroblast BJ/hTERT and epithelial RPE-340/hTERT cell lines were a kind gift from W.E. Wright [37] and were cultured in DMEM High Glucose (Gibco) and DMEM/F12 (1:1, Gibco) medium, respectively. All media were supplemented with 10% FCS (Biochrom GmbH, Berlin, Germany). Furthermore, 100 U/ml penicillin and 0.1 mg/ml streptomycin (Sigma-Aldrich, Merck Millipore, Darmstadt, Germany) was added to the tumor cell media. HCT116 and MCF-7 cells were authenticated by DNA fingerprinting (DSMZ, Braunschweig, Germany) and are regularly thawed (every 4-6 months) from these authentificated lot charge. All cell lines were routinely tested for mycoplasma contamination with the Venor®GeM detection kit (Minverva Biolabs, Berlin, Germany). Cells were driven into senescence by gamma-irradiation (γIR) with 10 (tumor cell lines) or 20 (primary cell lines) Gy using the Gulmay RS225 irradiation system (Xstrahl GmbH, Ratingen, Germany) at 150 kV/15 mA or by a treatment with 20 nM camptothecin. In all experiments, ‘senescent’ cells were used at day 7 after exposure to γIR or camptothecin, whereas ‘proliferating’ cells were seeded the day before treatment. The inhibitors wortmannin and BEZ235 as well as BYL-719 were obtained from LC Laboratories (Woburn, USA), whereas SAR405, PX-866, BAY80-6946, and CAL-101 were purchased from Biomol GmbH (Hamburg, Germany). The necroptosis inhibitors Nec-1s (Necrostatin-2 racemate) and GSK’872, the ferroptosis inhibitors Fer-1 (Ferrostatin-1) and CPX (ciclopirox olamine), and the fluorogenic caspase-3 substrate DEVD-AMC (N-acetyl-Asp-Glu-Val-Asp-aminomethylcoumarin) were also obtained from Biomol. Staurosporine, Torin-2 as well as propidium iodide (PI), X-gal (5-bromo-4-chloro-3-indolyl-β-D-galactopyranoside), TMRE (tetramethyl-rhodamine-ethylester), DTT, the protease inhibitors PMSF, aprotinin, leupeptin, pepstatin and the phosphatase inhibitors sodium ortho-vanadate and sodium pyrophosphate were all aquired from Sigma-Aldrich. From Selleckchem (Cologne, Germany) we purchased the anti-apoptotic Bcl-2-family member inhibitor Navitoclax (ABT-263), whereas the apoptosis inhibitor q-VD-OPH was from MP Biomedicals (Irvine, CA, USA). The proteasomal inhibitor MG-132 was from Enzo Life Sciences (Lausen, Germany), camptothecin from Merck Millipore, and DMSO from Applichem GmbH (Darmstadt, Germany).

### Antibodies

The beta-actin (#A5316; AB_476743) antibody was obtained from Sigma-Aldrich. From Calbiochem (Bad Soden, Germany) we purchased the monoclonal p53 (Ab-6) antibody (#OP43; AB_213405), whereas the p21 mouse or rabbit monoclonal antibodies used were from BD Biosciences (#556430; AB_396414; Heidelberg, Germany) or Cell Signaling (#2947, clone 12D1; AB_823586; Cell Signaling Technology Europe, B.V., Leiden, Netherlands), respectively. From Cell Signaling we also aquired the monoclonal cyclin D1 antibody (#2978, clone 92G2; AB_2259616), the polyclonal AKT antibody (#9272; AB_329827), as well as the monoclonal antibody directed against phospho-Ser473 of AKT (#4060, clone D9E; AB_2315049). The mouse monoclonal p16 (#sc-9968, clone 50.1; AB_628066) was purchased from Santa Cruz Biotechnology (Heidelberg, Germany). The infrared fluorescence-labeled secondary antibodies were from LI-COR Biosciences (Lincoln, Nebraska, USA).

### Colorimetric detection of senescence-associated beta-galactosidase activity in γ-irradiated cells

Cells were seeded and treated in 6-well plates. At the indicated times after γIR, cells were fixed in paraformaldehyde and glutaraldehyde containing buffer. After two washing steps with PBS, the samples were incubated at 37 °C for 8 (MCF-7, A549, BJ/hTERT, RPE-340/hTERT) to 16 (HCT116) hours with a staining buffer containing X-gal as a substrate and a suboptimal pH of 6.0 to analyze beta-galactosidase activity [57]. Staining was stopped by three washing steps with PBS before the samples were analyzed by microscopy. Senescent cells present themselves with an enlarged cellular and nuclear morphology and with a distinct blue color. All pictures were taken on an Axio Observer A1 microscope with the same 10× objective using the AxioVision Software (Carl Zeiss MicroImaging GmbH, Göttingen, Germany). For quantification purposes, 200 to 1100 cells (from 2–7 micrographs) for each condition and experiment were counted. The mean of senescence-associated beta-galactosidase positive cells (*i.e*., blue) cells ± SD per condition from at least three independent experiments is displayed in boxes in their corresponding micrographs.

### Screening of a natural compound library with a crystal violet-based cytotoxicity assay

An in-house compound library consisting of substances derived from marine sponges, endophytic fungi and higher plants was used for the screening approach [16, 17]. Cells were either seeded 8 days and gamma-irradiated 7 days (senescent) or seeded 1 day without irradiation exposure (proliferating) before they were incubated with the compounds (Fig. 1A). All compounds were employed in a serial dilution (30–0.1 µM) in a 96 well plate. After a 24 h treatment, dead and detached cells were removed by aspiration and the remaining living adherent cells stained with 0.5% crystal violet in 20% methanol as described elsewhere [58]. After drying, cells were solubilized in 200 µl 33% acetic acid for 30 min and the absorbtion at 595 nm detected in the Tecan M200 microplate reader (Mainz, Germany). Wells without any treatment served as the 100% control, whereas wells treated with 1% triton for 30 min before staining were used as 0% control. Every compound was tested in at least three independent experiments and every individual experiment was carried out in duplicates.

### Flow cytometric determination of cells with a loss of the mitochondrial membrane potential using TMRE staining

Proliferating and senescent cells were exposed to PX-866 and Navitoclax in 12-well plates. 24 h after treatment, the cells were stained by the addition of TMRE to a final concentration of 250 nM. After an incubation of 15 min at 37 °C, supernatants were collected into cold FACS tubes and the still adherent cells harvested by trypsinization. After detachment of the cells, they were transferred to their corresponding supernatant in the FACS tubes. The cells were pelleted by centrifugation and washed once in PBS before they were resuspended in 200 µl PBS. Subsequently, the cells were directly analyzed by flow cytometry using a FACS-Verse flow cytometer (BD Biosciences) and the FACSuite analysis software. As a negative control a sample of proliferating cells without added TMRE was used.

### Flow cytometric determination of cell cycle distribution by analysis of nuclear DNA content or of cell death by propidium iodide (PI) uptake

Cells were seeded and treated in 12-well plates. 24 h after treatment, supernatants were transferred into cold FACS tubes. The remaining living cells were detached by trypsinization and afterwards transferred into the corresponding supernatant-containing FACS tube. Cells were subsequently incubated for 15 min on ice to recover, before they were pelleted by centrifugation.

For cell cycle analyses, proliferating cells were directly resuspended in Nicoletti-buffer (0.1% sodium citrate, 0.1% Triton-X 100 and 50 µg/ml PI) and mixed thouroughly. After a 10 min incubation period on ice, the nuclear DNA content of the isolated nuclei was analyzed using a LSR Fortessa flow cytometer (BD Biosciences) and the FACSDiva analysis software. Nuclei containing G_1_, S, or G_2_/M DNA content were determined in a histogramm displaying PI-fluorescence intensity.

For cell death analyses, proliferating and senescent cells were directly resuspended in PI-buffer (2.5 µg/ml PI in PBS) and incubated for another 10 min, before they were analyzed by flow cytometry using the same setup as described above. Using a dot plot displaying the forward scatter (FSC) and PI-fluoresence intensity, the living, PI-negative population of each sample was determined. For each sample, at least 10,000 cells were analyzed.

In our hands, this type of cell death determination was not suitable for every cell line (e.g. HCT116, A549), because the trypsinization and pipetting procedure can result in false-positive and therefore highly over-estimated cell death numbers in senescent cell samples. Such cells at day 7 after senescence-inducing treatment are massively enlarged and do not react well to mechanical manipulation. Therefore, this kind of assay was only used to analyze the MCF-7 cell line because of its smaller cell size and greater resistance towards mechanical stress.

### Quantification of cell death by measuring LDH activity in the cell culture supernatant

Cell death induction results in the release of cytoplasmatic LDH into the cell culture supernatant. This was the preferred measurement of choice for death induction because no manipulation of the senescent cells themselves was required. The amount of released LDH was quantified by determining its specific activity in the supernatant with the cytotoxicity detection kit (LDH) from Roche (Mannheim, Germany) used according to the manufacturer’s instructions. In short, 100 µl cell-free culture supernatant aliquots of each sample were collected and mixed in a 96-well microtiter plate with an equal amount of the reaction mixture from the kit. LDH activity was quantified by measuring the absorbance at 490 nm using an reference absorbance of 690 nm in 3 min interverals for 30 min at room temperature in a Tecan M200 microplate reader. Data are either presented as arbritary units (AU) or as % of total LDH activity (calculated from control wells treated for 30 min with 1% Triton-X 100).

### Preparation of cell extracts and western blotting

Western Blotting was performed as described elsewhere [59]. In short, cell extracts were prepared by using the NP40 lysis buffer (1% NP-40, 50 mM Tris-HCL pH 7.4, 150 mM NaCl) supplemented with DTT (1 mM), the protease inhibitors aprotinin, leupeptin, pepstatin (all 10 µg/ml), PMSF (1 mM), and the phosphatase inhibitors sodium orthovanadate and sodium pyrophosphate (both at 1 mM). Protein concentrations were determined using the Bradford protein assay (BioRad, München, Germany). 30 µg of each sample were size-separated by SDS-PAGE and subsequently western blotted onto low-infrared-emitting PVDF membranes (Millipore, Schwalbach, Germany). Proteins were visualized by using corresponding primary antibodies and secondary infrared fluorochrome-labeled antibodies. The membranes were scanned using the LI-COR Odyssey FC and densitometrically quantified with the Image Studio V5.2 software.

### Fluorometric determination of caspase-3-like DEVDase activities in cellular extracts

Caspase activities were quantified as described [59]. In short, 50 µg cellular extract was mixed with caspase-substrate-buffer (50 mM HEPES pH 7.3, 100 mM NaCl, 10% Sucrose, 0.1% CHAPS) and 50 µM of the fluorogenic substrate DEVD-AMC in a black 96-well-microplate in a volume of 200 µl. Caspase activity was determined by measuring the generated fluorescence (Em.: 346 nm, Ex: 442 nm) in 10 min interverals for 3 h at 37 °C in a Tecan M200 microplate reader.

### Protein knockdown in proliferating and senescent cells using siRNAs

Cells were transfected with ON-TARGET-plus siRNA SMART-pools (Dharmacon, Horizon Discovery, Cambridge, UK) using DharmaFECT1 according to the manufacturer’s instructions. As a control, non-targeting SMART-pools were employed. In a 12-well plate, 50 pmol (proliferating) or 60 pmol (senescent) siRNA together with 2 or 3 µl DharmaFECT 1 in 1 ml medium were used, respectively. For senescent cells the transfection medium was changed after 24 h, whereas the transfected proliferating cells were evenly divided into several wells. After an additional 24 h, cells were treated with 3 µM PX-866 for another 24 h, before they were harvested and used for subsequent analyses. Although siRNA transfection resulted in an efficient protein knockdown, it resulted in an inevitable elevated background cytotoxiciy in senescent cells only.

### Statistical analyses

After checking the obtained data for normal distribution, a paired (if applicable) two-tailed student’s t-test (for two groups) or a one-way ANOVA followed by post-hoc Tukey´s multiple comparisons test (for three or more groups) were performed for all statistical analyses using GraphPad Prism 8. n.s. not significant, *p < 0.05, **p < 0.01, ***p < 0.001. Every analyzed experiment included negative and positive controls.

### Supplementary information

Supplementary Figure 1

Supplementary Figure 2

Supplementary Figure 3

Supplementary Table 1

Supplemental Material - Uncropped WB data

## Data Availability

The data generated in this study are available upon request from the corresponding author.

## References

[1] Gorgoulis V, Adams PD, Alimonti A, Bennett DC, Bischof O, Bishop C (2019). Cellular senescence: defining a path forward. Cell.

[2] Martinez-Zamudio RI, Robinson L, Roux PF, Bischof O (2017). SnapShot: cellular senescence pathways. Cell.

[3] Hernandez-Segura A, Nehme J, Demaria M (2018). Hallmarks of cellular senescence. Trends Cell Biol.

[4] Faget DV, Ren Q, Stewart SA (2019). Unmasking senescence: context-dependent effects of SASP in cancer. Nat Rev Cancer.

[5] Demaria M, Ohtani N, Youssef SA, Rodier F, Toussaint W, Mitchell JR (2014). An essential role for senescent cells in optimal wound healing through secretion of PDGF-AA. Dev Cell.

[6] Schmitt CA, Wang B, Demaria M (2022). Senescence and cancer - role and therapeutic opportunities. Nat Rev Clin Oncol.

[7] Baker DJ, Wijshake T, Tchkonia T, LeBrasseur NK, Childs BG, van de Sluis B (2011). Clearance of p16Ink4a-positive senescent cells delays ageing-associated disorders. Nature.

[8] Bussian TJ, Aziz A, Meyer CF, Swenson BL, van Deursen JM, Baker DJ (2018). Clearance of senescent glial cells prevents tau-dependent pathology and cognitive decline. Nature.

[9] Basu A (2022). The interplay between apoptosis and cellular senescence: Bcl-2 family proteins as targets for cancer therapy. Pharmacol Ther.

[10] Hu L, Li H, Zi M, Li W, Liu J, Yang Y (2022). Why senescent cells are resistant to apoptosis: an insight for senolytic development. Front Cell Dev Biol.

[11] Wang L, Lankhorst L, Bernards R (2022). Exploiting senescence for the treatment of cancer. Nat Rev Cancer.

[12] Zhu Y, Doornebal EJ, Pirtskhalava T, Giorgadze N, Wentworth M, Fuhrmann-Stroissnigg H (2017). New agents that target senescent cells: the flavone, fisetin, and the BCL-XL inhibitors, A1331852 and A1155463. Aging.

[13] Kovacovicova K, Skolnaja M, Heinmaa M, Mistrik M, Pata P, Pata I (2018). Senolytic cocktail dasatinib+quercetin (D+Q) does not enhance the efficacy of senescence-inducing chemotherapy in liver cancer. Front Oncol.

[14] Zhu Y, Tchkonia T, Pirtskhalava T, Gower AC, Ding H, Giorgadze N (2015). The Achilles’ heel of senescent cells: from transcriptome to senolytic drugs. Aging Cell.

[15] Petrova NV, Velichko AK, Razin SV, Kantidze OL (2016). Small molecule compounds that induce cellular senescence. Aging Cell.

[16] Deitersen J, Berning L, Stuhldreier F, Ceccacci S, Schlutermann D, Friedrich A (2021). High-throughput screening for natural compound-based autophagy modulators reveals novel chemotherapeutic mode of action for arzanol. Cell Death Dis.

[17] van Stuijvenberg J, Proksch P, Fritz G (2020). Targeting the DNA damage response (DDR) by natural compounds. Bioorg Med Chem.

[18] Ihle NT, Williams R, Chow S, Chew W, Berggren MI, Paine-Murrieta G (2004). Molecular pharmacology and antitumor activity of PX-866, a novel inhibitor of phosphoinositide-3-kinase signaling. Mol Cancer Ther.

[19] Sohn D, Essmann F, Schulze-Osthoff K, Janicke RU (2006). p21 blocks irradiation-induced apoptosis downstream of mitochondria by inhibition of cyclin-dependent kinase-mediated caspase-9 activation. Cancer Res.

[20] Martini H, Passos JF (2023). Cellular senescence: all roads lead to mitochondria. FEBS J.

[21] Zhu Y, Tchkonia T, Fuhrmann-Stroissnigg H, Dai HM, Ling YY, Stout MB (2016). Identification of a novel senolytic agent, navitoclax, targeting the Bcl-2 family of anti-apoptotic factors. Aging Cell.

[22] Adjemian S, Oltean T, Martens S, Wiernicki B, Goossens V, Vanden Berghe T (2020). Ionizing radiation results in a mixture of cellular outcomes including mitotic catastrophe, senescence, methuosis, and iron-dependent cell death. Cell Death Dis.

[23] Galluzzi L, Vitale I, Aaronson SA, Abrams JM, Adam D, Agostinis P (2018). Molecular mechanisms of cell death: recommendations of the Nomenclature Committee on Cell Death. Cell Death Differ 2018.

[24] Janicke RU, Sprengart ML, Wati MR, Porter AG (1998). Caspase-3 is required for DNA fragmentation and morphological changes associated with apoptosis. J Biol Chem.

[25] Janicke RU (2009). MCF-7 breast carcinoma cells do not express caspase-3. Breast Cancer Res Treat.

[26] Munoz-Espin D, Serrano M (2014). Cellular senescence: from physiology to pathology. Nat Rev Mol Cell Biol.

[27] Fukami J, Anno K, Ueda K, Takahashi T, Ide T (1995). Enhanced expression of cyclin D1 in senescent human fibroblasts. Mech Ageing Dev.

[28] Hemmings BA, Restuccia DF (2012). PI3K-PKB/Akt pathway. Cold Spring Harb Perspect Biol.

[29] Abbas T, Dutta A (2009). p21 in cancer: intricate networks and multiple activities. Nat Rev Cancer.

[30] Yosef R, Pilpel N, Papismadov N, Gal H, Ovadya Y, Vadai E (2017). p21 maintains senescent cell viability under persistent DNA damage response by restraining JNK and caspase signaling. EMBO J.

[31] Janicke RU, Sohn D, Essmann F, Schulze-Osthoff K (2007). The multiple battles fought by anti-apoptotic p21. Cell Cycle.

[32] Li Y, Dowbenko D, Lasky LA (2002). AKT/PKB phosphorylation of p21Cip/WAF1 enhances protein stability of p21Cip/WAF1 and promotes cell survival. J Biol Chem.

[33] Lee JY, Yu SJ, Park YG, Kim J, Sohn J (2007). Glycogen synthase kinase 3beta phosphorylates p21WAF1/CIP1 for proteasomal degradation after UV irradiation. Mol Cell Biol.

[34] Cross DA, Alessi DR, Cohen P, Andjelkovich M, Hemmings BA (1995). Inhibition of glycogen synthase kinase-3 by insulin mediated by protein kinase B. Nature.

[35] Mensah FA, Blaize JP, Bryan LJ (2018). Spotlight on copanlisib and its potential in the treatment of relapsed/refractory follicular lymphoma: evidence to date. Onco Targets Ther.

[36] Vanhaesebroeck B, Perry MWD, Brown JR, Andre F, Okkenhaug K (2021). PI3K inhibitors are finally coming of age. Nat Rev Drug Discov.

[37] Bodnar AG, Ouellette M, Frolkis M, Holt SE, Chiu CP, Morin GB (1998). Extension of life-span by introduction of telomerase into normal human cells. Science.

[38] Gorbunova V, Seluanov A, Pereira-Smith OM (2002). Expression of human telomerase (hTERT) does not prevent stress-induced senescence in normal human fibroblasts but protects the cells from stress-induced apoptosis and necrosis. J Biol Chem.

[39] Rodier F, Coppe JP, Patil CK, Hoeijmakers WA, Munoz DP, Raza SR (2009). Persistent DNA damage signalling triggers senescence-associated inflammatory cytokine secretion. Nat Cell Biol.

[40] Thorpe LM, Yuzugullu H, Zhao JJ (2015). PI3K in cancer: divergent roles of isoforms, modes of activation and therapeutic targeting. Nat Rev Cancer.

[41] Yu M, Chen J, Xu Z, Yang B, He Q, Luo P (2023). Development and safety of PI3K inhibitors in cancer. Arch Toxicol.

[42] Sanchez-Vega F, Mina M, Armenia J, Chatila WK, Luna A, La KC (2018). Oncogenic Signaling Pathways in The Cancer Genome Atlas. Cell.

[43] Chen YH, Wei MF, Wang CW, Lee HW, Pan SL, Gao M (2015). Dual phosphoinositide 3-kinase/mammalian target of rapamycin inhibitor is an effective radiosensitizer for colorectal cancer. Cancer Lett.

[44] Gil del Alcazar CR, Hardebeck MC, Mukherjee B, Tomimatsu N, Gao X, Yan J (2014). Inhibition of DNA double-strand break repair by the dual PI3K/mTOR inhibitor NVP-BEZ235 as a strategy for radiosensitization of glioblastoma. Clin Cancer Res.

[45] Prevo R, Deutsch E, Sampson O, Diplexcito J, Cengel K, Harper J (2008). Class I PI3 kinase inhibition by the pyridinylfuranopyrimidine inhibitor PI-103 enhances tumor radiosensitivity. Cancer Res.

[46] Mukherjee B, Tomimatsu N, Amancherla K, Camacho CV, Pichamoorthy N, Burma S (2012). The dual PI3K/mTOR inhibitor NVP-BEZ235 is a potent inhibitor of ATM- and DNA-PKCs-mediated DNA damage responses. Neoplasia.

[47] Chen JS, Zhou LJ, Entin-Meer M, Yang X, Donker M, Knight ZA (2008). Characterization of structurally distinct, isoform-selective phosphoinositide 3’-kinase inhibitors in combination with radiation in the treatment of glioblastoma. Mol Cancer Ther.

[48] Kuger S, Graus D, Brendtke R, Gunther N, Katzer A, Lutyj P (2013). Radiosensitization of glioblastoma cell lines by the dual PI3K and mTOR Inhibitor NVP-BEZ235 depends on drug-irradiation schedule. Transl Oncol.

[49] Ogawara Y, Kishishita S, Obata T, Isazawa Y, Suzuki T, Tanaka K (2002). Akt enhances Mdm2-mediated ubiquitination and degradation of p53. J Biol Chem.

[50] Child ES, Mann DJ (2006). The intricacies of p21 phosphorylation: protein/protein interactions, subcellular localization and stability. Cell Cycle.

[51] Vasudevan KM, Barbie DA, Davies MA, Rabinovsky R, McNear CJ, Kim JJ (2009). AKT-independent signaling downstream of oncogenic PIK3CA mutations in human cancer. Cancer Cell.

[52] Lien EC, Dibble CC, Toker A (2017). PI3K signaling in cancer: beyond AKT. Curr Opin Cell Biol.

[53] Althubiti M, Rada M, Samuel J, Escorsa JM, Najeeb H, Lee KG (2016). BTK modulates p53 activity to enhance apoptotic and senescent responses. Cancer Res.

[54] An S, Cho SY, Kang J, Lee S, Kim HS, Min DJ (2020). Inhibition of 3-phosphoinositide-dependent protein kinase 1 (PDK1) can revert cellular senescence in human dermal fibroblasts. Proc Natl Acad Sci USA.

[55] Sieben CJ, Sturmlechner I, van de Sluis B, van Deursen JM (2018). Two-step senescence-focused cancer therapies. Trends Cell Biol.

[56] Bunz F, Dutriaux A, Lengauer C, Waldman T, Zhou S, Brown JP (1998). Requirement for p53 and p21 to sustain G2 arrest after DNA damage. Science.

[57] Debacq-Chainiaux F, Erusalimsky JD, Campisi J, Toussaint O (2009). Protocols to detect senescence-associated beta-galactosidase (SA-betagal) activity, a biomarker of senescent cells in culture and in vivo. Nat Protoc.

[58] Sohn D, Totzke G, Essmann F, Schulze-Osthoff K, Levkau B, Janicke RU (2006). The proteasome is required for rapid initiation of death receptor-induced apoptosis. Mol Cell Biol.

[59] Sohn D, Peters D, Piekorz RP, Budach W, Janicke RU (2016). miR-30e controls DNA damage-induced stress responses by modulating expression of the CDK inhibitor p21WAF1/CIP1 and caspase-3. Oncotarget.

