# Supplementary figures and images for: Combined inhibition of class 1-PI3K-alpha and delta isoforms causes senolysis by inducing p21WAF1/CIP1 proteasomal degradation in senescent cells
Source: Cell Death Dis. 2024 May 29;15(5):373. doi: 10.1038/s41419-024-06755-x (PMC11136996; doi:10.1038/s41419-024-06755-x)

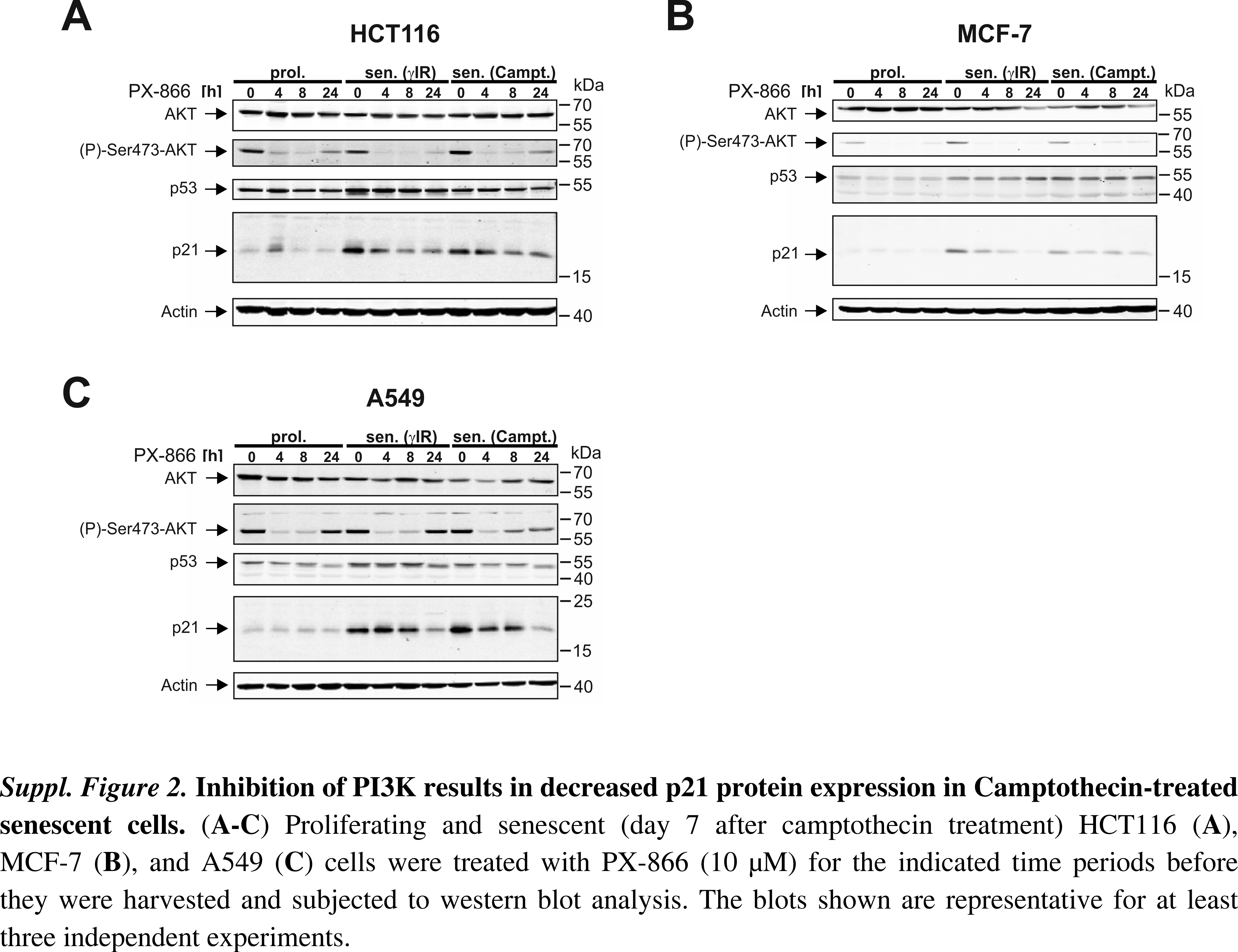

Supplement: Supplementary file 2 — Supplementary Figure 2 [file 41419_2024_6755_MOESM2_ESM.tif]

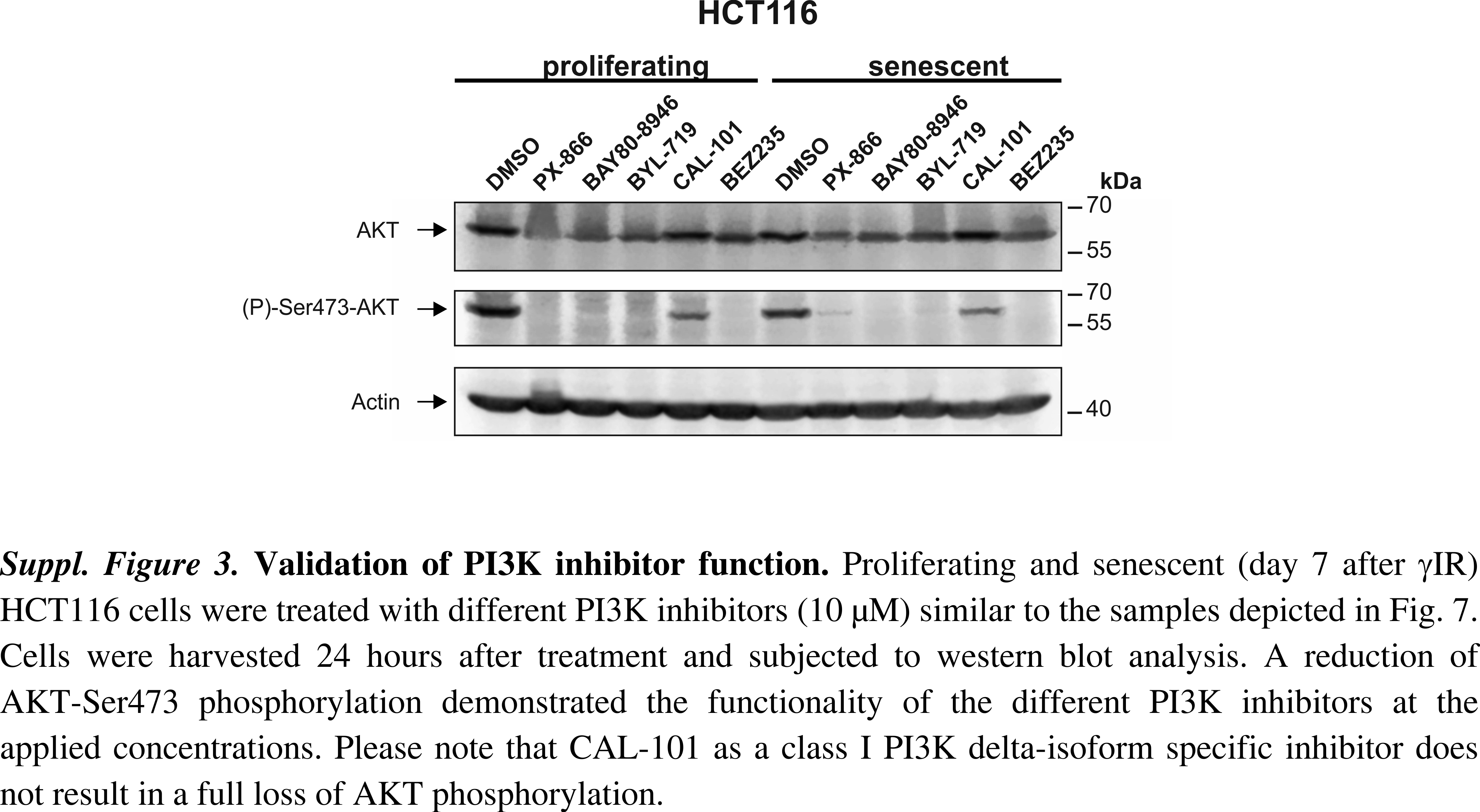

Supplement: Supplementary file 3 — Supplementary Figure 3 [file 41419_2024_6755_MOESM3_ESM.tif]
