# Supplementary material for: Combined inhibition of class 1-PI3K-alpha and delta isoforms causes senolysis by inducing p21WAF1/CIP1 proteasomal degradation in senescent cells
Source: Cell Death Dis. 2024 May 29;15(5):373. doi: 10.1038/s41419-024-06755-x (PMC11136996; doi:10.1038/s41419-024-06755-x)
Supplement: Supplementary file 4 — Supplementary Table 1 [file 41419_2024_6755_MOESM4_ESM.docx]

***Suppl. Table 1: Classification of all tested compounds based on their impact on proliferating and senescent HCT116 cells.***

| **Phenotype** | **Compound** |
| --- | --- |
| **No effect** | dibromohemibastadin-1; oxime ester; (-) α–bisabolol; nor‑bromhemibastadin; cinnamic acid; visnagin; oximic acid; phomoxanthone A; 5,5'-dibromohemibastadin-1; ester-Br_2_; aeroplysinin; berberine hemisulfate; W493 B; cholest-5-en-3β-ol/(22E, 24S)-24-methylcholesta-5,22-dien-3β-ol; 24-ethylcholesta-5-en-3β-ol; benzyl nitrile; debromohymenialdisine; aloesin; hydroxydienoic acid; bakuchiol; tetrahydroxybostricin; isobava chalcone; alternariol; roquefortine C; Chlorogenic acid; aloe emodin; midpacamide; embeurecol B; Br_2_-hydroxyethylamide; corynesidone A; 7-O-methylaloeresin A; 4-(4,5-dibromo-1-methyl-1H-pyrrole-2-carboxamido) butanoic acid; ergosterol; citrinin; aloeresin A; demethoxyencecalin; 3,5-Dibromo-2-hydroxy-4–methoxyphenylacetonitrile; catechin; sclerotiorin; (-) ageloxime D; aranorosinol B; sekikaic acid; cyclopenol; homosekikaic acid; N-methyl-4,5-dibromopyrrole-2-carboxylic acid; meleagrin; kojic acid; 2,2-dimethylchroman-3,6-diol; (E)-methyl-3-(-4-methoxyphenoxy) propenoate; hydroxysydonic acid; alternariol monomethyl ether; Br-phenethylamide; Br-isobutylamide; phenol A acid; xanthorrhizol; waolic acid; 4,5-dibromo-1H-pyrrol-2–carboxyamide; hymenidin; hexylamide; Br_2_-tyrosine; Br_2_-hexylamide; 4,5-dibromo-1H-pyrrole-2–carboxylic acid ethyl ester; dihydrogeodin; 3,5-dibromo-1H-pyrrole-2-carboxylic acid; mauritamide B; indole-3-carboxylic acid; dasyclamide; viridicatin; scorzodihydrostilbene B; anomalin A; tetrahydroxystilbeneglucoside; 4′-O-methyl norhomosekikaic acid; Br-histamide; Br_2_-phenethylamide; 18-dehydroxycytochalasin H; 4´,5,7–trimethoxydihydroflavonol; theonellapeptolide Ie; piperine; lutein; 3,4,5-Tri-O-methylgallic acid butyl ester; 3-O-methylgallic acid butyl ester; dibromohydroxyphakellin; (+) agelasidine C; orientin; tiliroside; Br_2_-histamide; kaempferitrin; flavomannin A; alternarienoic Acid; cytochalasin D derv.; Br_2_-tryptamide; euparin; 2,3,4-trimethyl-5,7-dihydroxy-3-dihydrobenzofuran; pinocembrin; scorzodihydrostilbene A; feralolide; N-trans-feruoyltyramine; aerophobin 2; aposphaerin A; W493 A; (S) - (-) rhodoptilometrin; N-methyl-4,5-dibromopyrrole-2-methylcarboxylate; paxillin; tryptamide; dienone dimethoxyketal; cyclohexylamide; 3-O-methylgallic acid methyl ester; 3-O-methylgallic acid propyl ester; genestein; 3,5-dicaffeoylquinic acid; resacetophenone; histamide; bionectriamide A; imiquimod; corynesidone C; L-tryptophane; altenusin; 9,21–didehydroryanodine; Br-cyclohexylamide; isobutylamide; 3,4,5-O-trimethyl-gallate; lasiodiplodin; 6-methoxycomaparvin-5-methyl ether; 5-(3, 5-dibromo-4-) (2-oxooxazolidin-5yl)methoxy)phenyl)oxazolidin-2 –one; kaempferol-3-O-β-D-glucopyranosyl(1---4) α-L-rhamnopyranosyl-7-O-α-L–rhamnopyranoside; cerebroside D; warfarin; myrocin A; 3,5-Dibromo-2-benzoyloxy-4– methoxyphenylacetonitrile; callyaerin F; 3-methoxybutyl gallate; agelanin B; eupatoriumchromene 1; 4-methoxybenzoic acid; isoferulic acid methylester; syringic acid; 4-bromopyrrole-2–carboxamide; hyperoside; 2-hydroxy-4–methoxyphenylacetonitrile; acteoside; 1,3-dihydro-4-hydroxy-1(1-hydroxyethyl)-3-oxoisobenzofuran-5-carboxylic acid; mauritamide C; atromentine. |
| **Cytotoxic for proliferating cells** | macrosporin; (-) matairesinol; manzamine A; neobavaisoflavone; (-) arctigenin; stemphyperylenol; avarone; skyrin; (+) avarol; kuanoniamine D; isofistularin-3; ilimaquinone; cladosporin; alterporriol D; 5-epi-nakijiquinone Q; viriditoxin; luffariellolide. |
| **Cytotoxic for proliferating and senescent cells** | helenalin; agelasine D; aerothionin; (+) aeroplysinin-1; WLIP; 4,6-dibromo-2-(2´,4´-dibromophenoxy)phenol; 3,4,6-tribromo-2-(2´,4´-dibromophenoxy)phenol; isovitexin; dienone; enniatin B; altersolanol A; aranorosin; enniatin A1; 8-OH-manzamine A; citreodrimene B; scopularide A. |
| **Senolytic** | wortmannin A; kahalalide F |
