# Supplementary material for: Combined inhibition of class 1-PI3K-alpha and delta isoforms causes senolysis by inducing p21WAF1/CIP1 proteasomal degradation in senescent cells
Source: Cell Death Dis. 2024 May 29;15(5):373. doi: 10.1038/s41419-024-06755-x (PMC11136996; doi:10.1038/s41419-024-06755-x)

Supplemental Raw Data
Western Blot

Neuwahl et al. 2024

Figure 5A

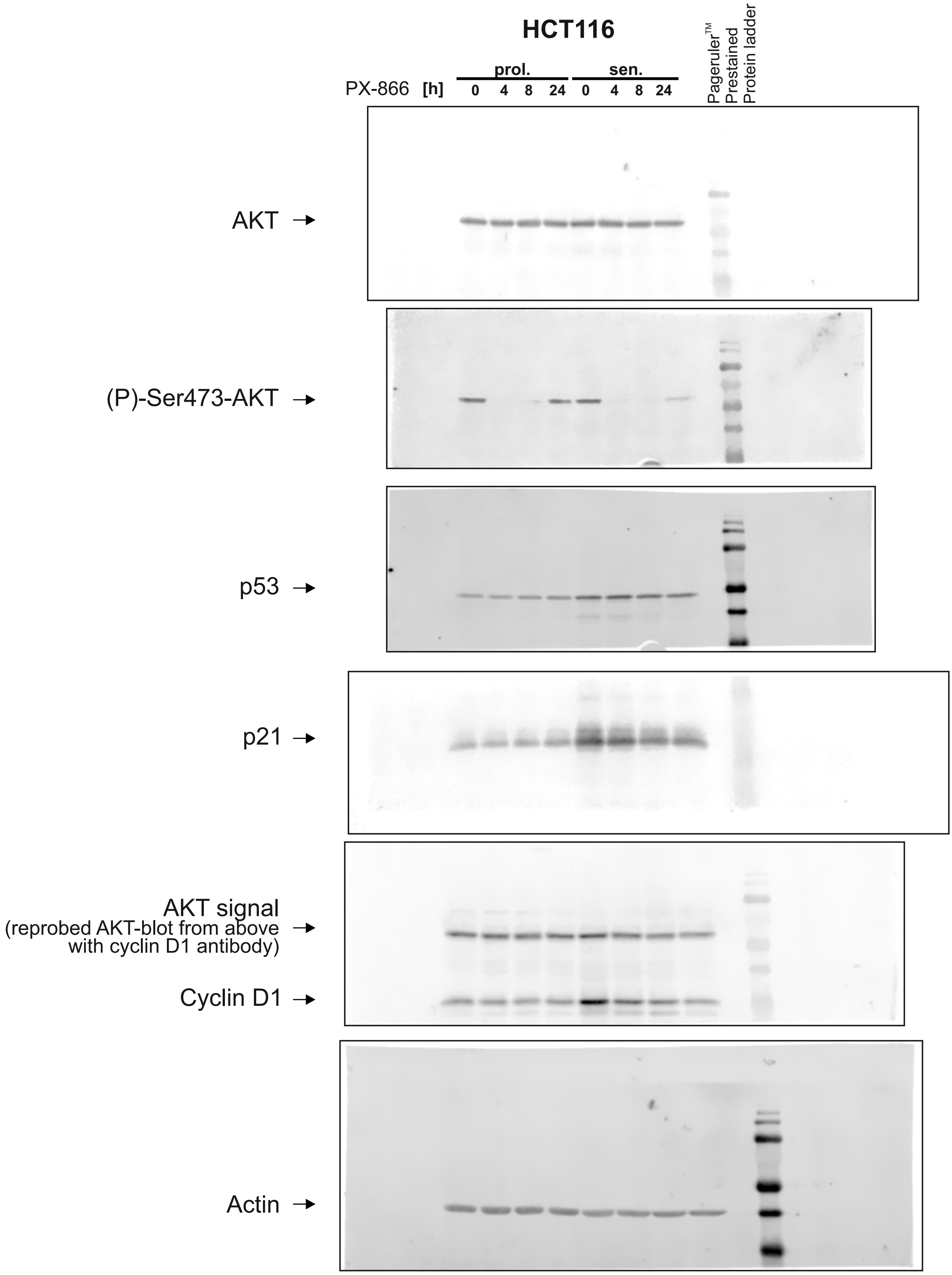

Figure 5B

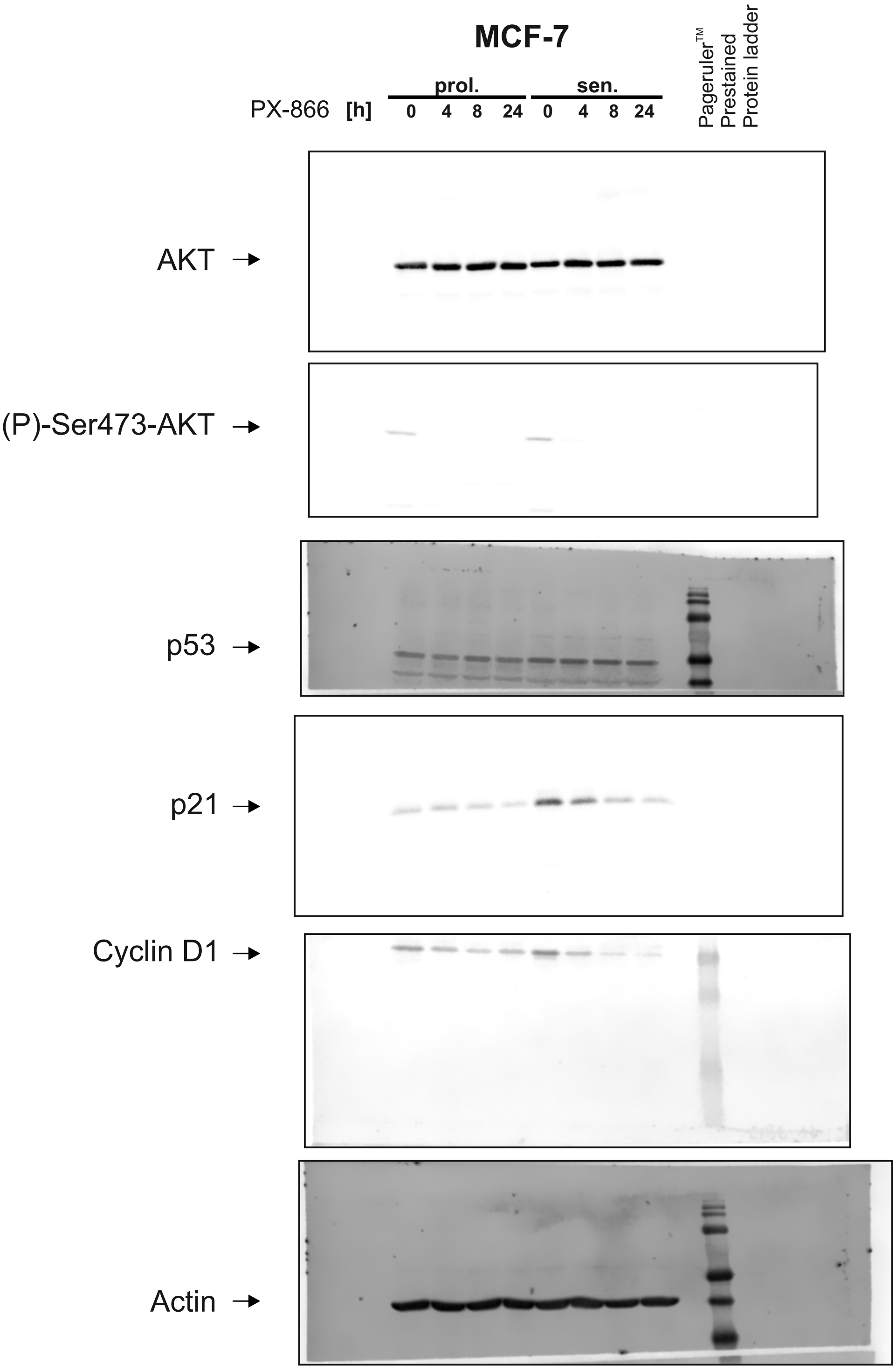
MCF-7
prol.
sen.
PX-866 [h]
0 4 8 24 0 4 8 24
Pageruler™ Prestained Protein ladder
AKT
(P)-Ser473-AKT
p53
p21
Cyclin D1
Actin

Figure 5C

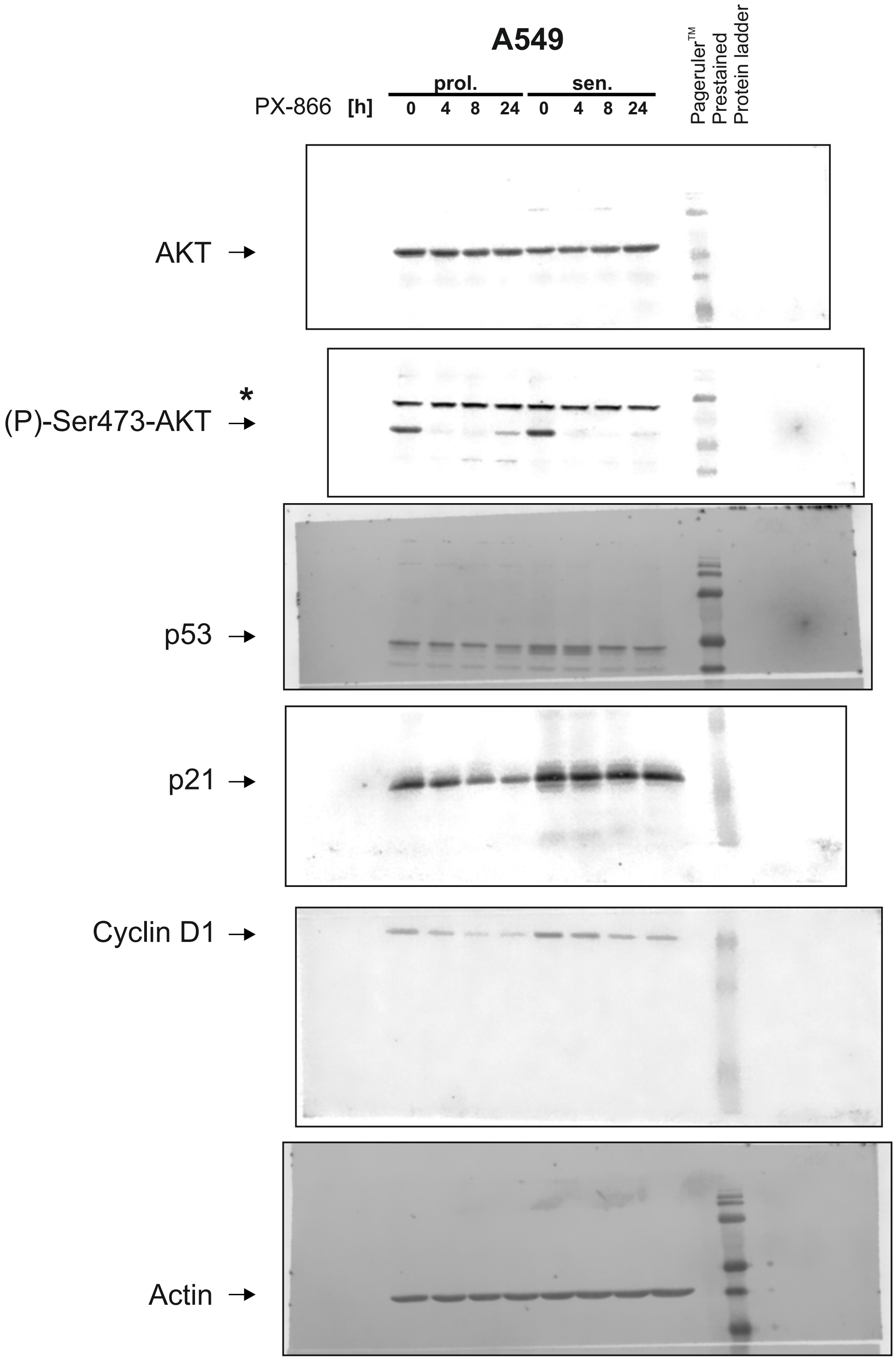
A549
prol.
sen.
PX-866 [h]
0
4
8
24
0
4
8
24
Pageruler™ Prestained Protein ladder
AKT
*
(P)-Ser473-AKT
p53
p21
Cyclin D1
Actin

Figure 6A

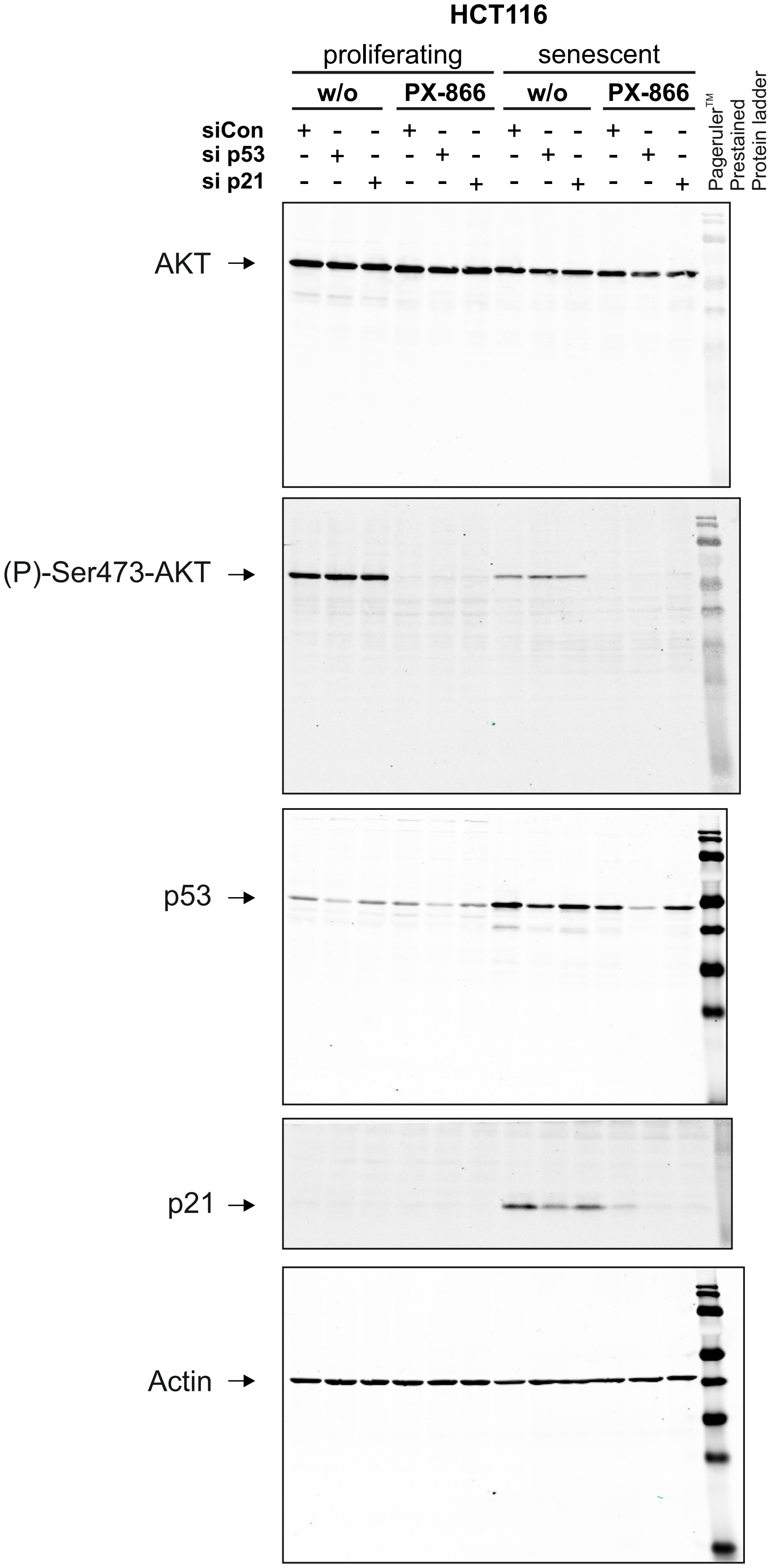
HCT116
proliferating
senescent
w/o
PX-866
w/o
PX-866
siCon
si p53
si p21
Pageruler™ Prestained Protein ladder
AKT
(P)-Ser473-AKT
p53
p21
Actin

Supplemental Raw Data
Western Blot

Neuwahl et al. 2024

Figure 6C

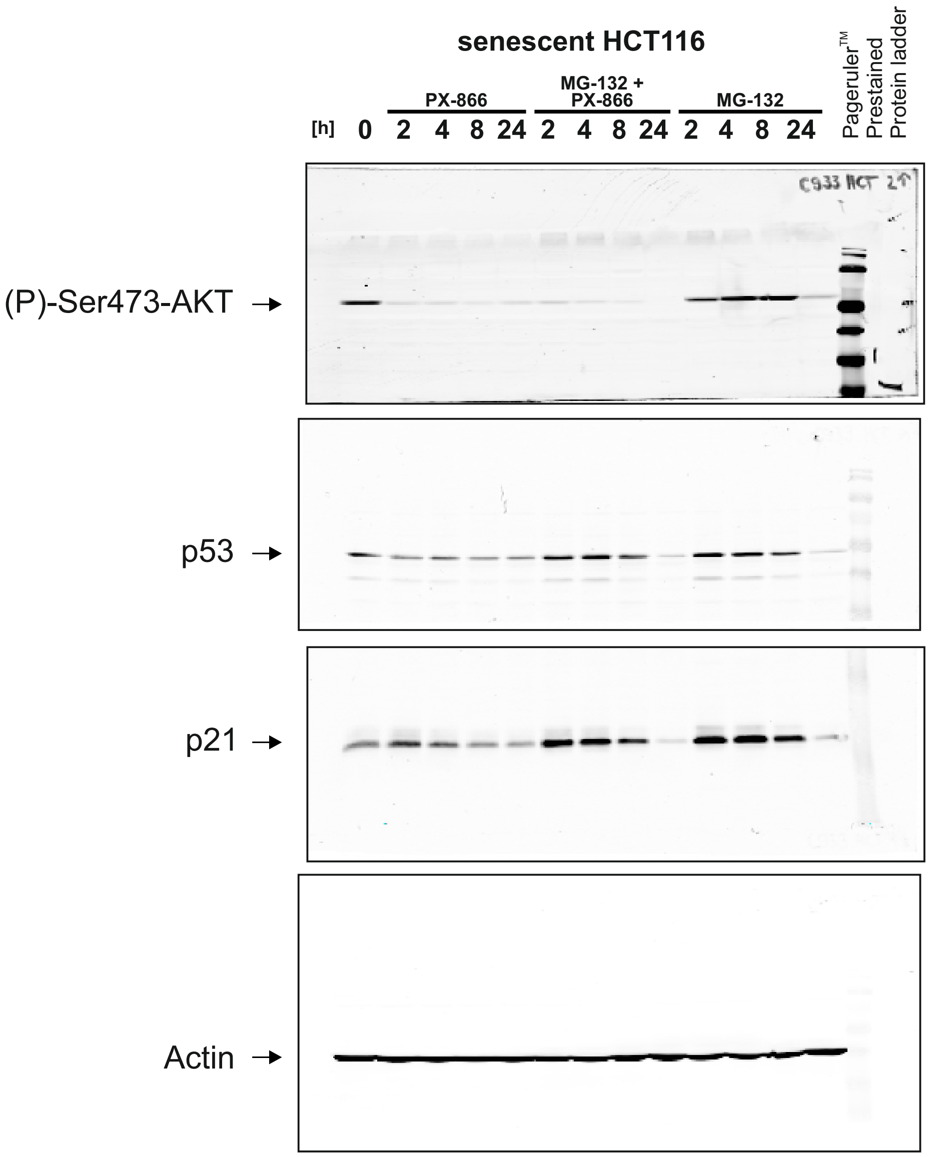

Supplemental Raw Data
Western Blot

Neuwahl et al. 2024

Figure 6E

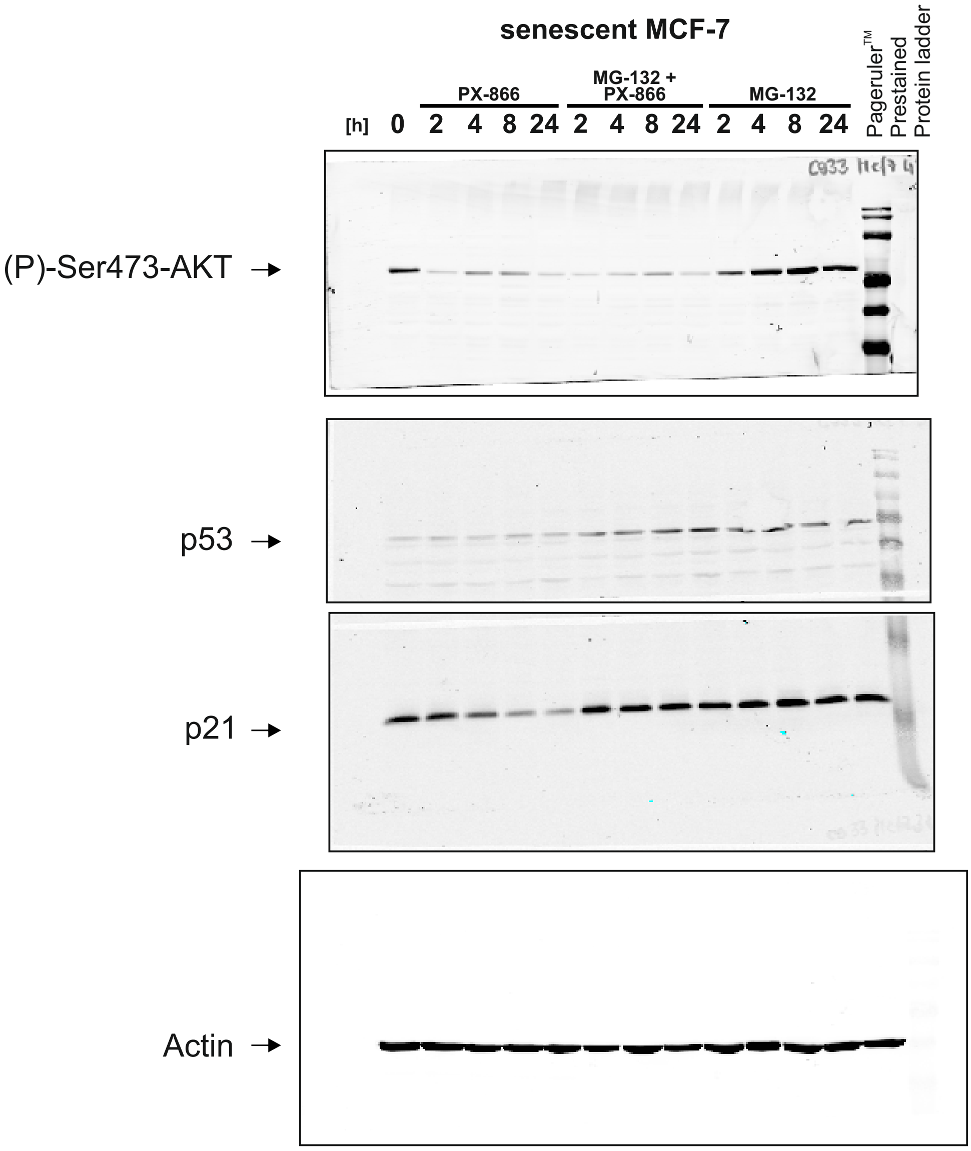

Supplemental Raw Data
Western Blot

Neuwahl et al. 2024

Figure 8B

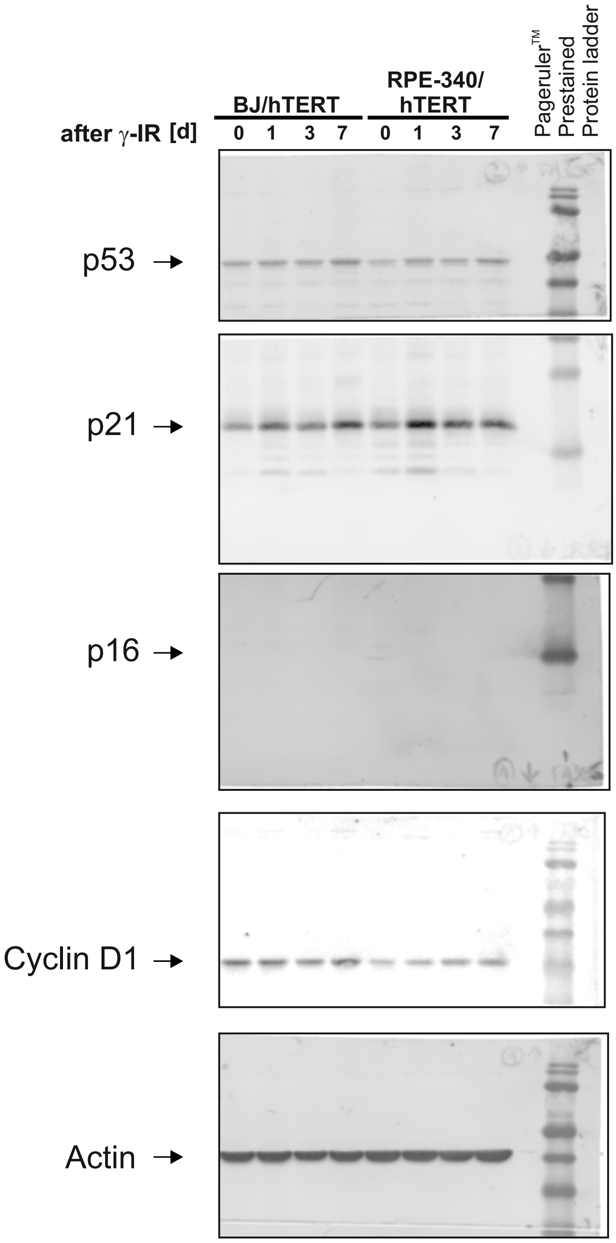

Supplemental Raw Data Western Blot

Neuwahl et al. 2024

Suppl. Figure 2A

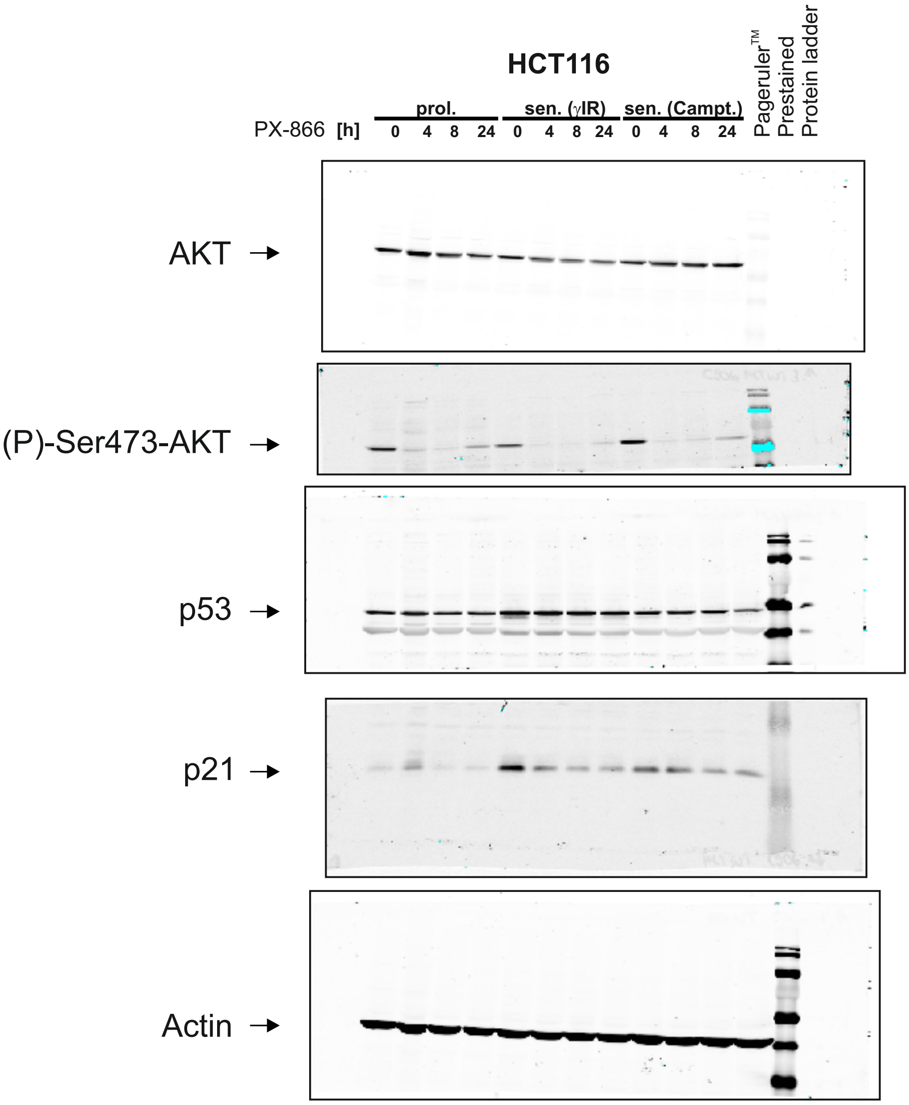

Supplemental Raw Data
Western Blot

Neuwahl et al. 2024

Suppl. Figure 2B

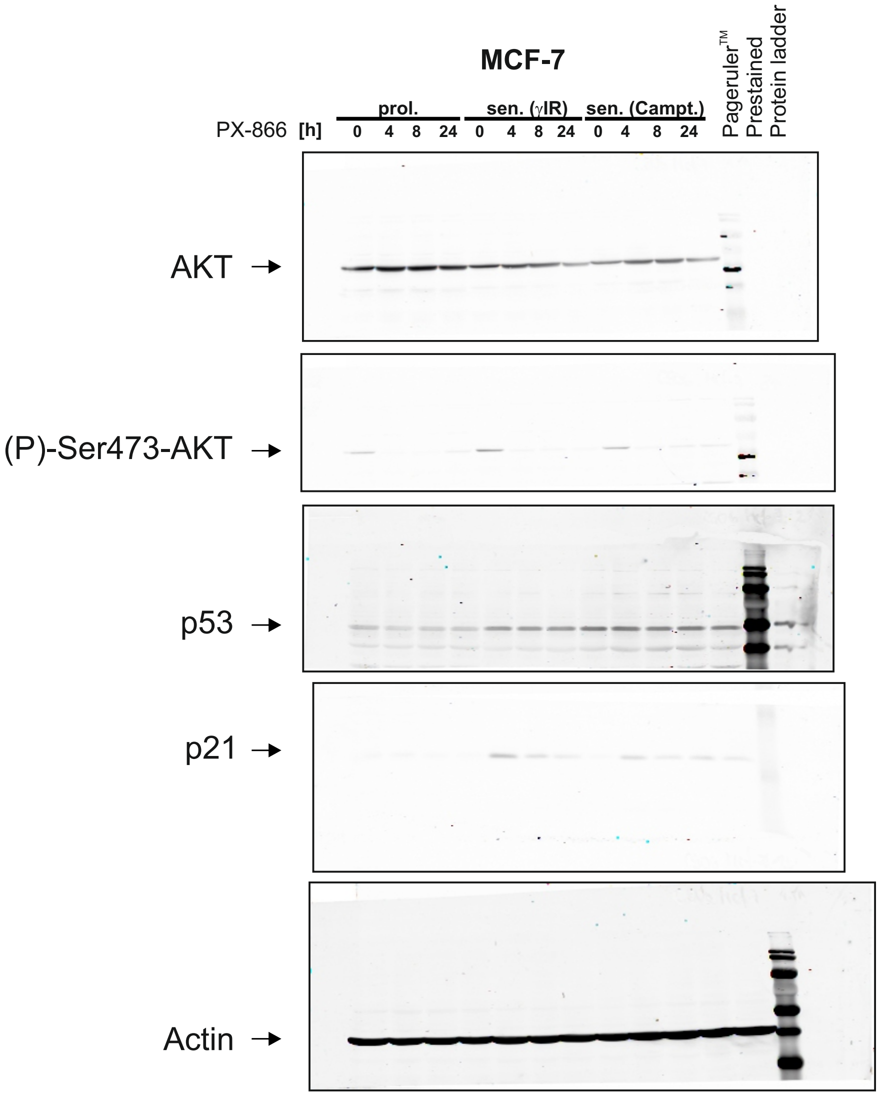

Suppl. Figure 2C

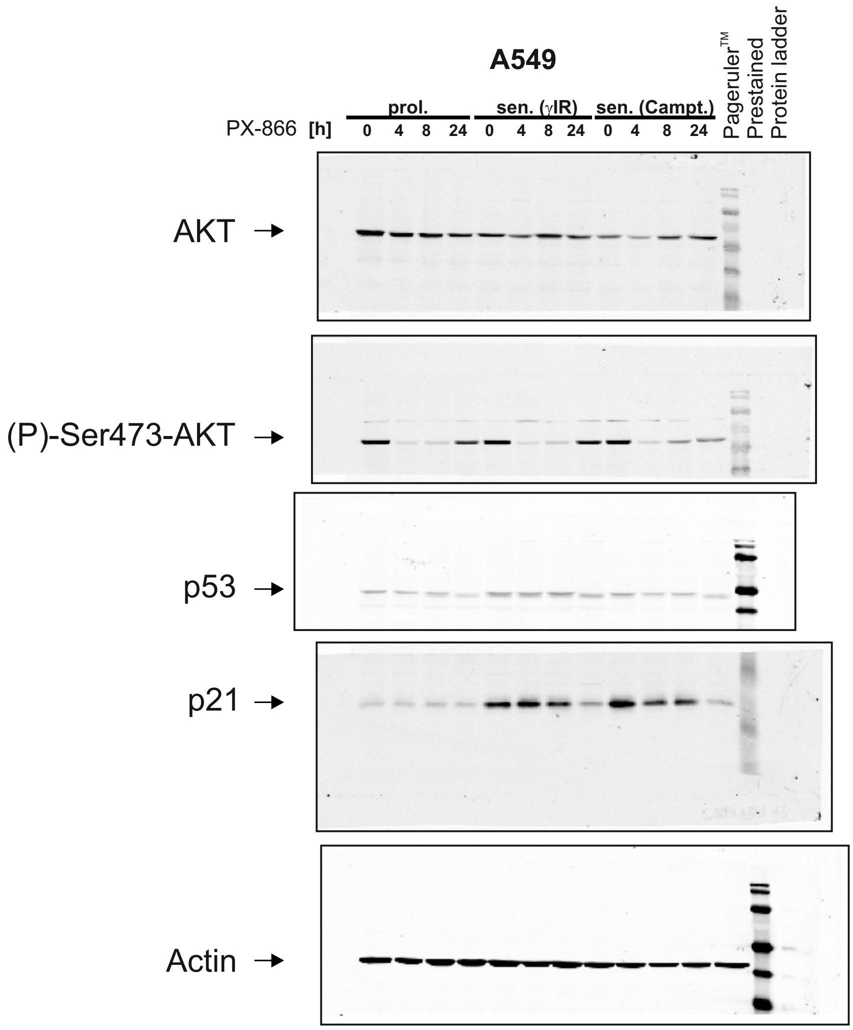

Supplemental Raw Data
Western Blot

Neuwahl et al. 2024

Suppl. Figure 3

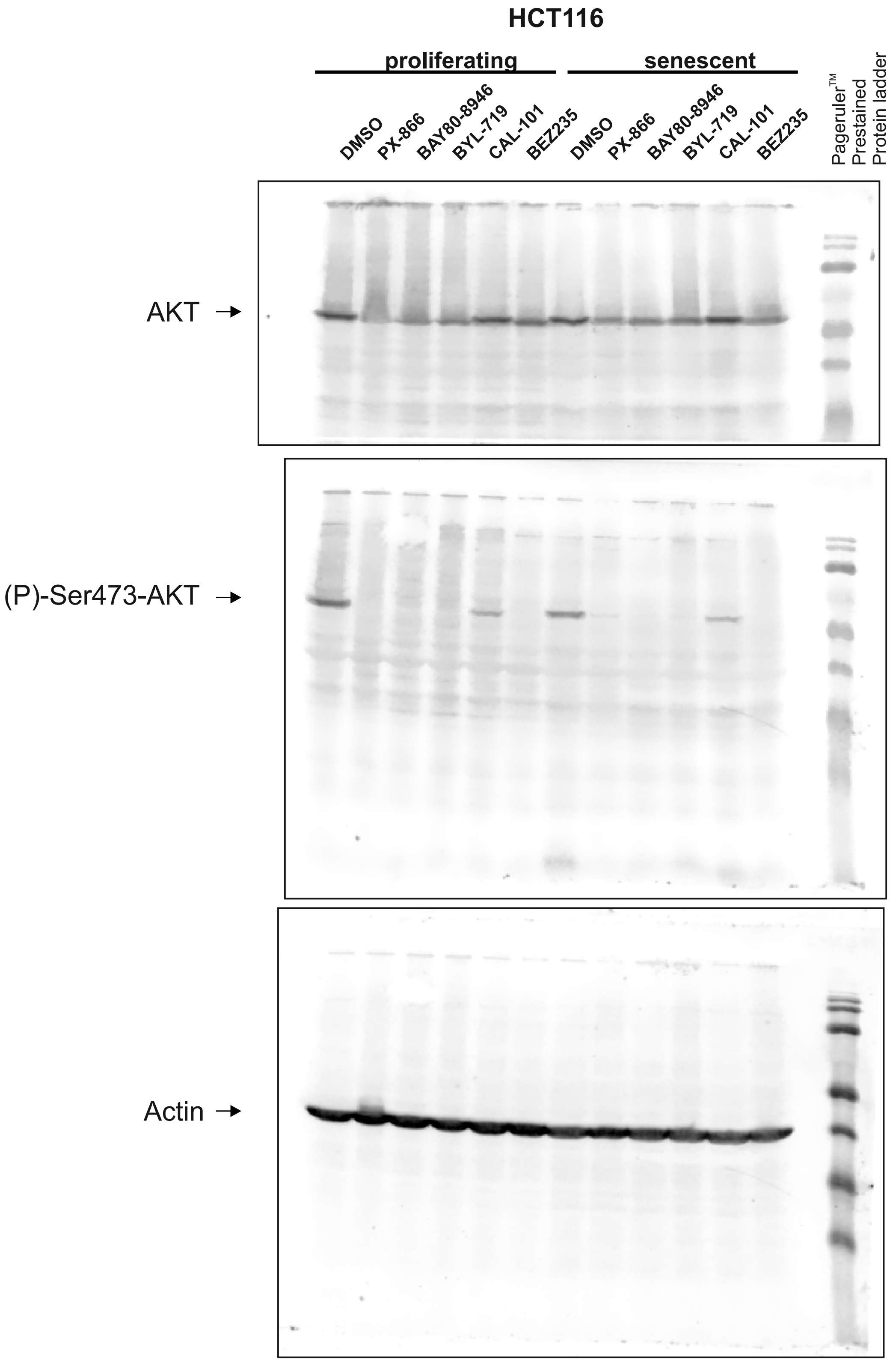

Supplement: Supplementary file 5 — Supplemental Material - Uncropped WB data [file 41419_2024_6755_MOESM5_ESM.pdf]
